# SETD2-dependent H3K36me3 plays a critical role in epigenetic regulation of the HPV31 life cycle

**DOI:** 10.1371/journal.ppat.1007367

**Published:** 2018-10-12

**Authors:** Dipendra Gautam, Bryan A. Johnson, Michelle Mac, Cary A. Moody

**Affiliations:** 1 Lineberger Comprehensive Cancer Center, University of North Carolina at Chapel Hill, Chapel Hill, North Carolina, United States of America; 2 Department of Microbiology and Immunology, University of North Carolina at Chapel Hill, Chapel Hill, North Carolina, United States of America; Penn State University School of Medicine, UNITED STATES

## Abstract

The life cycle of HPV is tied to the differentiation status of its host cell, with productive replication, late gene expression and virion production restricted to the uppermost layers of the stratified epithelium. HPV DNA is histone-associated, exhibiting a chromatin structure similar to that of the host chromosome. Although HPV chromatin is subject to histone post-translational modifications, how the viral life cycle is epigenetically regulated is not well understood. SETD2 is a histone methyltransferase that places the trimethyl mark on H3K36 (H3K36me3), a mark of active transcription. Here, we define a role for SETD2 and H3K36me3 in the viral life cycle. We have found that HPV positive cells exhibit increased levels of SETD2, with SETD2 depletion leading to defects in productive viral replication and splicing of late viral RNAs. Reducing H3K36me3 by overexpression of KDM4A, an H3K36me3 demethylase, or an H3.3K36M transgene also blocks productive viral replication, indicating a significant role for this histone modification in facilitating viral processes. H3K36me3 is enriched on the 3’ end of the early region of the high-risk HPV31 genome in a SETD2-dependent manner, suggesting that SETD2 may regulate the viral life cycle through the recruitment of H3K36me3 readers to viral DNA. Intriguingly, we have found that activation of the ATM DNA damage kinase, which is required for productive viral replication, is necessary for the maintenance of H3K36me3 on viral chromatin and for processing of late viral RNAs. Additionally, we have found that the HPV31 E7 protein maintains the increased SETD2 levels in infected cells through an extension of protein half-life. Collectively, our findings highlight the importance of epigenetic modifications in driving the viral life cycle and identify a novel role for E7 as well as the DNA damage response in the regulation of viral processes through epigenetic modifications.

## Author summary

High-risk HPVs are associated with multiple human cancers, most notably cervical cancer. Understanding mechanisms by which HPV co-opts cellular pathways to replicate could identify potential therapeutic targets. The HPV genome is associated with histones in a manner similar to that of cellular DNA, but how histone modifications influence the viral life cycle is not well understood. Here, we demonstrate that high-risk HPV positive cells exhibit elevated levels of SETD2 in an E7-dependent manner. SETD2 places the trimethyl mark on H3K36 (H3K36me3) and we have found that SETD2 as well as H3K36me3 are necessary for productive viral replication and splicing of late viral RNAs upon epithelial differentiation. H3K36me3 is present on the HPV31 genome suggesting that SETD2 regulates viral processes through the recruitment of effector proteins to H3K36me3 on viral DNA. In addition, we have found that HPV31 maintains H3K36me3 on viral chromatin and regulates splicing of viral RNAs through activation of the ATM DNA damage response, which is also required for required productive viral replication. Overall, these findings advance our understanding of how the viral life cycle is epigenetically regulated and identify a novel role for the DNA damage response in facilitating viral processes.

## Introduction

Human papillomaviruses (HPVs) are small, circular, double-stranded DNA viruses that have a genome of approximately 8 kilobases. More than 200 types of HPV have been identified and are categorized based on their dominant site of infection; either the cutaneous or mucosal epithelium [1]. Mucosal HPVs are further divided into high and low-risk types based on their potential to induce transformation [2]. The high-risk types, of which there are ~15, are the causative agents of cervical cancer [3]. In addition, high-risk HPV types are also associated with other anogenital cancers, as well as an increasing number of head and neck cancers [4]. HPV-associated cancers are driven by the two main oncoproteins E6 and E7, which do not exhibit enzymatic function, but work primarily through protein-protein interactions, affecting cellular signaling pathways to provide a replication-competent environment [5, 6].

The life cycle of HPV is intimately linked to the differentiation of its host cell, the keratinocyte, and consists of three phases of replication [7, 8]. HPV infects the actively dividing basal cells of the stratified epithelium upon exposure through a microwound. Upon entry into the nucleus, the viral genome undergoes a transient amplification to 50–100 episomal copies per cell in a process termed establishment replication. In undifferentiated basal cells, the viral genome is maintained at a low copy number by replicating once per cell cycle along with cellular DNA [9, 10]. In these cells, early viral genes (E1, E2, E6, E7) are expressed at a low level from the early promoter (p97 for HPV31), which is located upstream of the E6 open reading frame in the upstream regulatory region (URR) [11, 12]. Epithelial differentiation triggers the productive phase of the viral life cycle, which results in activation of the late promoter (p742 for HPV31) and high levels of the replication proteins E1 and E2 to drive viral genome amplification to hundreds to thousands of copies per cell [11–16]. E4 and E5 are also expressed at high levels upon differentiation and contribute to productive replication through mechanisms that are not well understood [17, 18]. Transcripts encoding the capsid proteins L1 and L2 are only generated upon differentiation, such that virion assembly is restricted to the uppermost layers of the epithelium [19, 20]. Normally, epithelial differentiation results in an exit from the cell cycle. However, the limited coding capacity of the viral genome renders HPV reliant on cellular factors for replication. HPV supports productive replication by subverting key pathways that regulate host cell replication, in turn maintaining differentiating cells active in the cell cycle [6]. This is accomplished in large part through the E7 protein, which pushes differentiating cells back into the cell cycle through its ability to target members of the retinoblastoma family of tumor suppressors (pRb, p107, p130) for degradation [21, 22]. In addition, E7 sustains a replication-competent environment upon differentiation by maintaining activation of the ATM- and ATR-dependent DNA damage response (DDR) pathways, which are essential for productive viral replication [23–29].

The HPV genome is histone-associated in infected cells as well as in viral particles [30–32]. The chromosomal organization of HPV genomes is similar to that of cellular chromatin and is thought to be regulated by histone-based modifications [33]. Histone tails are subject to extensive post-translational modifications, including acetylation, phosphorylation, and methylation, which can occur at particular genic regions (e.g. enhancer, promoter, gene body) [34]. Histone marks are dynamic and occur as a balance between enzymes that deposit the mark (writers) and other enzymes that remove the mark (erasers). Epigenetic readers, which are often part of larger, multi-subunit protein complexes, bind directly to the histone mark through a particular domain, serving as effector proteins [35]. It has become clear that these histone modifications play fundamental roles in most cellular processes that require access to DNA [36, 37]. During the viral life cycle, the early and late promoters of HPV31 exhibit an active conformation, characterized by acetylated H3 and H4 as well as dimethyl H3K4, suggesting that viral transcription is coordinated by histone modifications [38]. In support of this, the Tip60 histone acetyltransferase, the SIRT1 deacetylase and the chromatin-binding protein Brd4 have been implicated in viral transcription as well as replication [39–41]. In addition, histone modifications associated with DNA repair (e.g. γH2AX) are found on HPV31 genomes [42]. The E6 and E7 oncoproteins are well established to induce epigenetic changes in cellular chromatin by affecting the expression or activity of numerous epigenetic modifiers, including histone acetyltransferases, histone deacetylases, histone methyltransferases and histone demethylases [40, 43]. However, how E6/E7 modulation of host epigenetic machinery regulates viral chromatin and the impact of these epigenetic changes on the viral life cycle remains largely uncharacterized.

SETD2 is a methyltransferase that interacts with the Ser2 phosphorylated C-terminal domain of RNA polymerase II (RNA pol II) and places the trimethyl mark on H3K36 (H3K36me3) during transcription elongation [44, 45]. H3K36me3 is thus a mark of active transcription and increases along gene bodies peaking at the 3’ end [46, 47]. Through the recruitment of numerous readers of the H3K36me3 mark, SETD2 regulates multiple cellular processes, including modulation of chromatin structure and maintenance of transcription initiation through nucleosome remodeling, as well as alternative splicing through recruitment of splicing factors [48–52]. In addition, SETD2 is associated with DNA replication and repair through the recruitment of factors to H3K36me3 involved in homologous recombination (HR) as well as mismatch repair [53–55]. Alternative splicing is a key mechanism by which HPV regulates viral gene expression and ensures maximal protein production from a compact genome [56]. In addition, previous studies from our lab and others have demonstrated that HR repair factors are bound to HPV DNA and are required for productive viral replication [41, 57]. Since SETD2-regulated cellular processes through H3K36me3 are also central to successful completion of the HPV life cycle, we wanted to determine if SETD2-mediated H3K36me3 contributes to epigenetic regulation of HPV replication.

In this study, we have found that high-risk HPV positive cells exhibit high levels of SETD2 protein in an E7-dependent manner that are required for productive replication upon differentiation and also contribute to episomal maintenance in undifferentiated cells. H3K36me3 is enriched on the early region of the HPV31 genome in a SETD2-dependent manner. Interestingly, we have found that ATM kinase activity contributes to maintenance of H3K36me3 on the viral genome, identifying a novel role for DDR activation in epigenetic regulation of the viral life cycle. Depletion of H3K36me3 by SETD2 knockdown or inhibition of ATM blocks productive viral replication and alters splicing of late RNAs, suggesting that the recruitment of H3K36me3 readers to HPV chromatin is critical to the viral life cycle. Intriguingly, we have found that HPV31 increases the stability of SETD2 protein in a manner dependent on E7’s Rb binding domain. These studies not only provide insight into how chromatin modifications affect viral processes, but also identify a novel role for E7 in the epigenetic regulation of the HPV life cycle through increasing levels of the epigenetic modifier SETD2.

## Results

### HPV positive cells exhibit increased levels of the SETD2 methyltransferase

**Fig 1:**
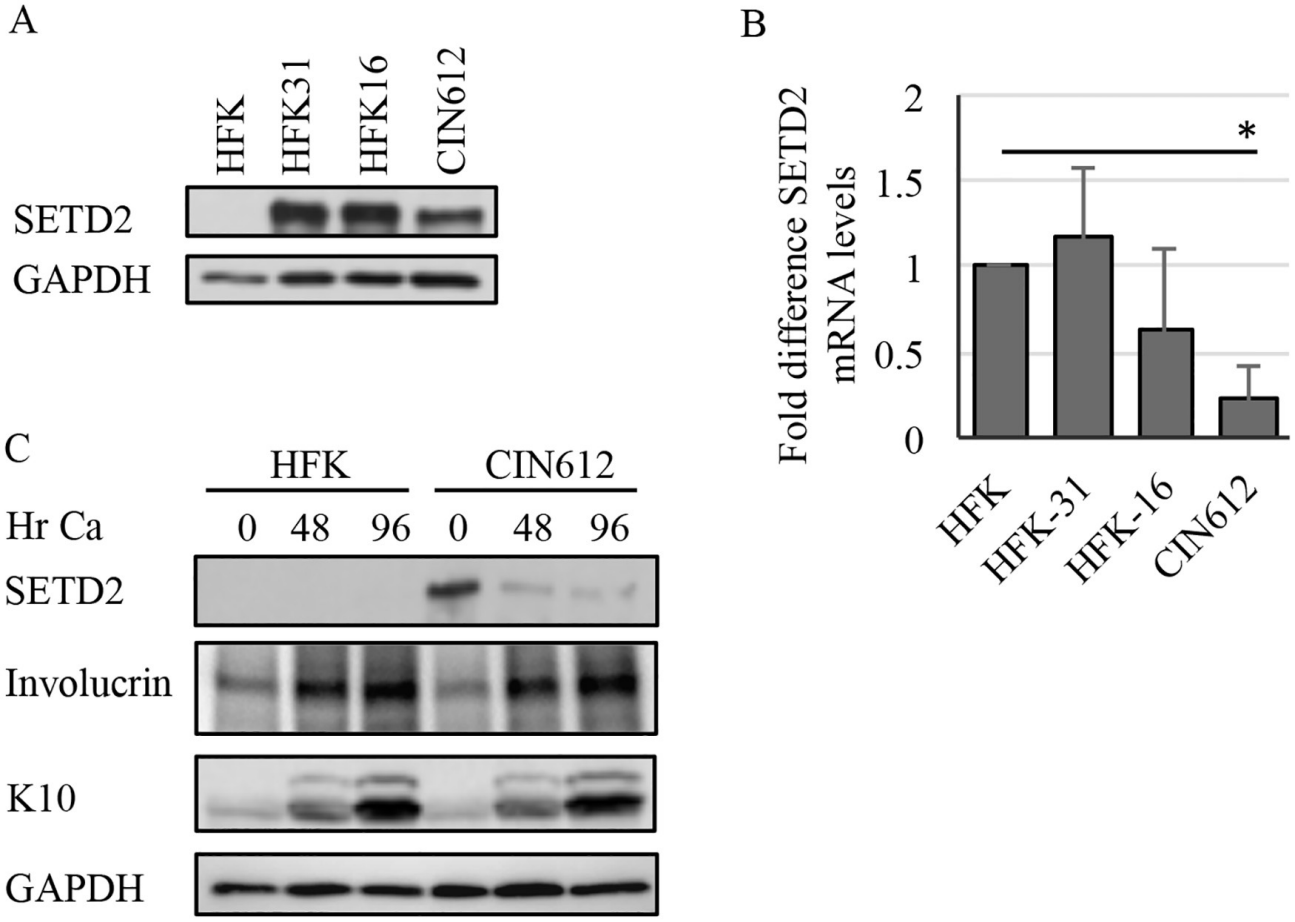
SETD2 protein levels are increased in HPV positive cells. (A) Lysates were harvested from undifferentiated human foreskin keratinocytes (HFKs), HFKs stably maintaining HPV16 (HFK-16) or HPV31 (HFK-31) genomes as well as HPV31 positive CIN612 cells, and western blot analysis was performed using an antibody to SETD2. GAPDH served as loading control. (B) Total RNA was extracted from undifferentiated HFKs, HFK-31, HFK-16 and CIN612 cells and quantitative RT-PCR was performed using primers specific to SETD2. Fold change was calculated using 2^−ΔΔCT^ method. Shown is the fold-change relative to HFKs, which is set to 1. The values represent the average of three independent experiments. Error bars represent means +/- standard error. Statistics were assayed using a student’s t test. *p≤ .05. (C) Lysates were harvested from undifferentiated (T0) HFKs and CIN612 cells, as well as after differentiation in high calcium medium (48, 96hr), and western blot analysis was performed using an antibodies to SETD2 as well as involucrin and keratin 10 (K10) as differentiation controls. p84 serving as a loading control,. (A, C) Shown is a representative image of three independent experiments. Ca = calcium.

To determine if SETD2 could play a role in the HPV life cycle, we first examined SETD2 levels in HPV positive cells. For these studies, we used human foreskin keratinocytes (HFKs) stably transfected with HPV31 (HFK-31) or HPV16 (HFK-16) genomes. We also used CIN612 9E (CIN612) cells, which are derived from a CIN1 cervical lesion and maintain HPV31 genomes episomally [11]. As shown in Fig 1A, HPV31 (HFK-31, CIN612) as well as HPV16 positive cells (HFK-16) exhibited higher levels of SETD2 protein compared to uninfected HFKs. While we did observe some variation in SETD2 levels based on the HFK background, the levels of SETD2 in the HFK-31, HFK-16 and CIN612 cells were consistently higher than that found in HFKs. Interestingly, in contrast to substantial differences in protein levels, the transcript levels of SETD2 were similar between the HFKs, HFK-31 and HFK-16 cells (Fig 1B). Surprisingly, the SETD2 mRNA levels were significantly lower in the CIN612 cells compared to the HFKs, despite high levels of protein. These results indicate that SETD2 protein levels are increased post-transcriptionally in HPV positive cells. To determine if the increased levels of SETD2 protein are maintained upon differentiation, we examined SETD2 protein levels in HFKs and CIN612 cells grown in high calcium medium for 48hr and 96hr, which is a commonly used method to induce the productive phase of the viral life cycle. As shown in Fig 1C, although SETD2 levels decreased in CIN612 cells upon differentiation, they were still consistently present at higher levels than in HFKs. Involucrin and keratin 10 (K10) were examined as markers of epithelial differentiation.

### SETD2 levels are increased in HPV positive cells in an E7-dependent manner

**Fig 2:**
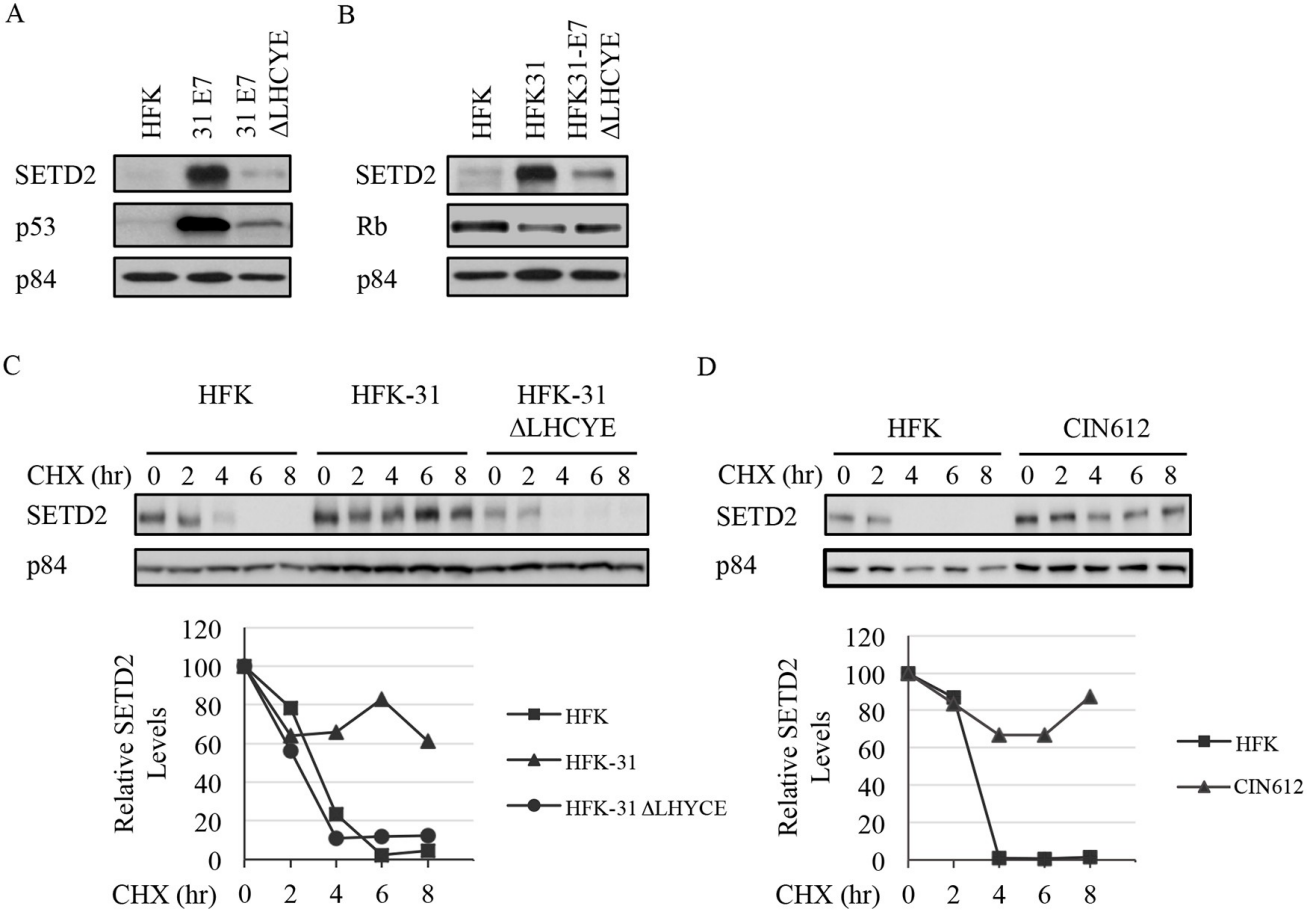
SETD2 is increased post-transcriptionally in HPV31 positive cells in an E7-dependent manner. Whole cell lysates were harvested from (A) uninfected HFKs, HFKs retrovirally transduced and stably expressing either wild-type E7 or E7 containing a deletion of the Rb binding domain (^Δ^LHCYE) as well as (B) HFKs, HFKs stably maintaining wild-type HPV31 genomes (HFK-31) or genomes containing the E7 ^Δ^LHCYE mutation (HFK-31 ^Δ^LHCYE). Western blot analysis was performed using antibodies to SETD2, (A) p53 and (B) pRb, with p84 as a loading control. Shown is a representative image of three independent experiments. (C) HFKs, HFKs containing wild-type HPV31 genomes (HFK-31) or genomes containing a mutation in E7’s Rb binding domain (^Δ^LHCYE), as well as (D) HFKs and CIN612 were treated with 50 ug/ml cycloheximide over an 8hr time course. Lysates were harvested at the indicated time points and western blot analysis was performed using an antibody to SETD2, as well as p84 as a loading control. Graphed are the relative protein levels at each time point normalized to GAPDH, with T0 for each cell line set to 100. Densitometry was performed using Biorad ImageLab 5.0 software. Data shown is representative of three independent experiments.

The HPV oncoprotein E7 plays a crucial role in maintaining higher levels of many cellular factors required for the viral life cycle, several of which require E7’s Rb binding domain [25, 58]. Furthermore, E7 induces the expression and/or affects the activity of several DNA/chromatin modifying enzymes [40, 43]. To determine if SETD2 levels are increased in an E7-dependent manner, we examined SETD2 protein levels in HFKs retrovirally transduced and expressing either wild-type HPV31 E7, or E7 containing a mutation in the Rb binding domain (^Δ^LHCYE), both of which are stably expressed [59]. As shown in Fig 2A, E7-expressing cells had substantially increased levels of SETD2 protein compared to HFKs, and this phenotype was lost in cells containing the ^Δ^LHCYE mutant. We found that co-expression of E6 with E7 did not alter the levels of SETD2, indicating that E7 is primarily responsible for the increase in SETD2 (S1 Fig). To determine if E7 is necessary for the elevated levels of SETD2 protein observed in the context of HPV infection, we generated HFKs that maintain either wild type HPV31 genomes or genomes containing the E7 ^Δ^LHCYE Rb binding mutation. We, and others, have shown that HFKs containing ^Δ^LHCYE mutant viral genomes are maintained episomally, but exhibit a defect in productive viral replication as well as reduced episome copy number over time [25, 59]. As shown in Fig 2B, similar to E7 expression alone, the increase in SETD2 levels we observed in HFK-31 cells compared to HFKs was lost in HFK-31 ^Δ^LHCYE cells. Overall, these studies demonstrate that the increase in SETD2 protein in HPV positive cells occurs in an E7-dependent manner, requiring E7’s Rb binding domain.

SETD2 is normally present at low levels due to rapid turnover by proteasome-dependent degradation by the SPOP ubiquitin ligase [60]. Previous studies from our lab demonstrated that E7 increases the protein half-life of several DNA repair factors required for viral replication [25]. To determine if E7 increases SETD2 at the level of protein stability, we examined the half-life of SETD2 in HFKs and HFK-31 cells using cycloheximide to block protein synthesis. As shown in Fig 2C, while the half-life of SETD2 protein in HFKs was approximately 3hr, the half-life was extended to greater than 8hrs (the longest time point measured) in HFK-31 cells. The increase in SETD2 protein half-life was lost in HFK-31 ^Δ^LHCYE cells, indicating that E7 increases the protein stability of SETD2 in a manner dependent on its Rb binding domain. CIN612 cells exhibited a similar increase in SETD2 protein half-life as the lab-generated HFK-31 lines (Fig 2D). Taken together, these results demonstrate that E7, through its Rb binding domain, post-transcriptionally regulates the levels of SETD2 in HPV positive cells through an increase in protein stability.

### SETD2 is necessary for HPV31 replication

**Fig 3:**
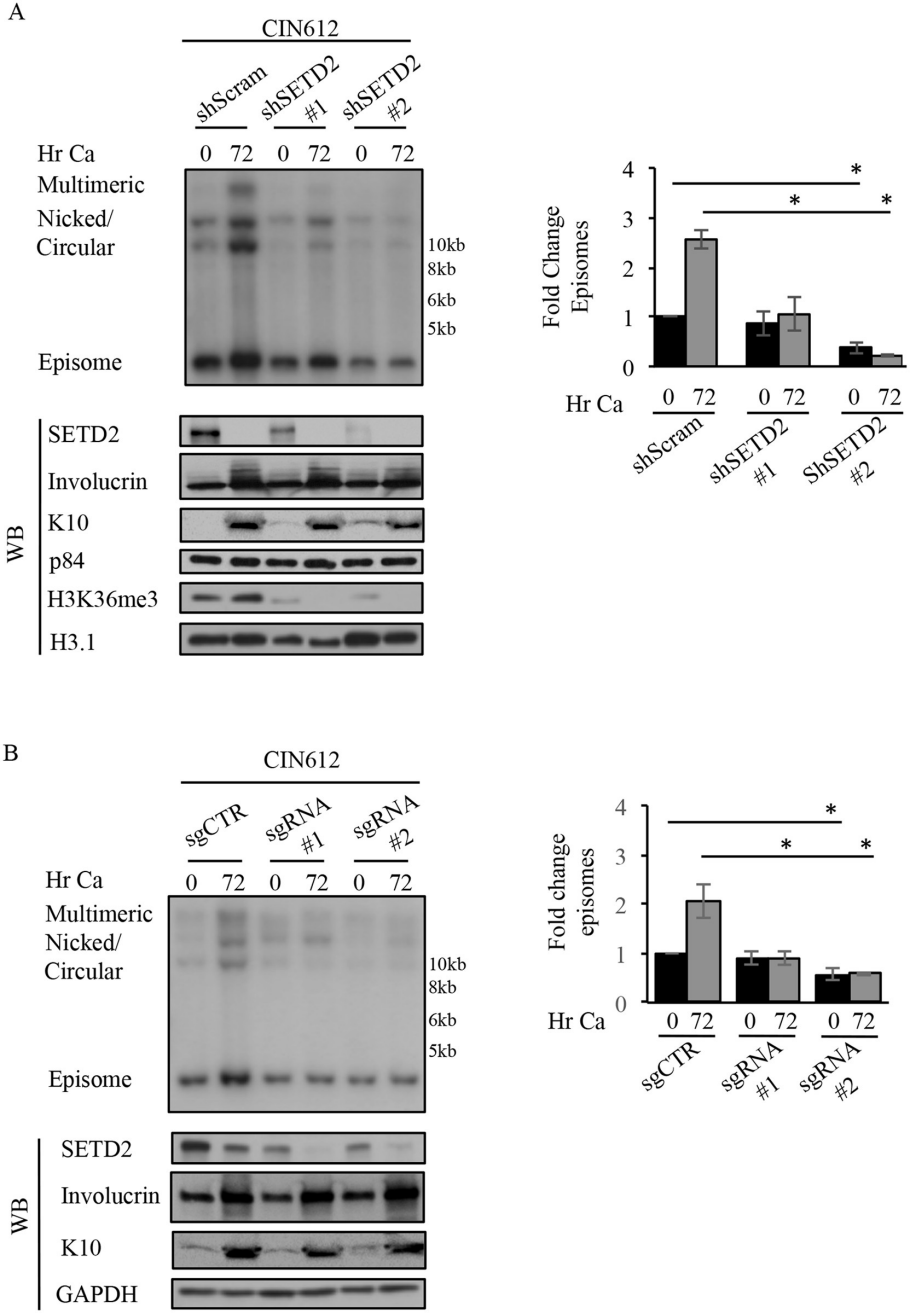
SETD2 is necessary for productive viral replication. (A) CIN612 cells were transiently transduced with either a scramble control shRNA (shScram) or one of two SETD2 shRNAs (shSetd2#1 and shSetd2#2) for 72hrs. At this time, DNA and protein were either harvested as an undifferentiated (T0) sample, or cells were grown in high calcium medium to induce differentiation (72hr). DNA was digested with BamHI, which does not cut the viral genome, and Southern blot analysis was performed using the HPV31 genome as a probe. Lysates harvested at the indicated time points were analyzed by immunoblotting to demonstrate the decrease in SETD2 and H3K36me3 upon shRNA-mediated knockdown. Involucrin and K10 were used as markers of differentiation, and p84 and histone H3.1 (H3.1) served as loading controls. (B) CIN612 cells were transduced with lentivirus expressing either control guide RNAs (sgCTR) or guide RNAs targeting SETD2 (sgSETD2 #1 and sgSETD2 #2) and selected with puromycin. Following selection, DNA and protein were harvested from the heterogenous population of cells. DNA was digested with BamHI (non-cutter) and Southern blot analysis performed using the HPV31 genome as a probe. Western blot analysis was performed to examine the levels of SETD2, involucrin and K10 as differentiation controls, with GAPDH as a loading control. (A, B) Fold change in episome copy number for SETD2 knockdown using shRNAs as well as guide RNAs was determined by performing densitometry of episomal bands from three independent experiments using ImageJ software. Shown is the fold change relative to shScram T0 (A) and sgCTR T0 (B), which are set to one. Error bars represent means +/- standard error. Statistics were assayed using a student’s t test. *p≤ .05. WB = western blot. Ca = calcium.

To determine if the increased SETD2 levels are important for the HPV life cycle, we examined the impact of SETD2 depletion on viral replication in undifferentiated and differentiated cells using small hairpin RNAs (shRNA). CIN612 cells were transduced with lentiviruses expressing a control shRNA (shScram) or two different SETD2-specific shRNAs (shSETD2 #1 or shSETD2 #2). 72 hours post-transduction, undifferentiated cells were either harvested (T0), or differentiated in high calcium medium for 72hr. SETD2 is the sole methyltransferase that places the trimethyl mark on H3K36me3 [45], and as expected SETD2 knockdown resulted in a global decrease in H3K36me3 (Fig 3A). In addition, SETD2 depletion resulted in a dose-dependent decrease in episomal copy number in undifferentiated cells that correlated with the efficiency of SETD2 knockdown, with shRNA #1 and #2 decreasing episome copy number by ~12% and ~60%, respectively. SETD2 knockdown with both shRNAs resulted in a block in productive viral replication upon differentiation (Fig 3A). Importantly, levels of the differentiation specific markers involucrin and K10 were not affected by loss of SETD2, indicating that the defect in viral genome amplification was not an indirect effect of blocking cellular differentiation. In addition, using SETD2 shRNA #2, we found that SETD2 depletion minimally altered the levels of cellular factors involved in cell cycle regulation in undifferentiated or differentiated cells, including cyclin A and cyclin E (S-phase cyclins) and CDK2 (cyclin-dependent kinase 2), mitotic cyclin B and CDK1 as well as the Cdc25c phosphatase (S2A Fig). We also observed minimal impact on levels of the cellular replication protein RPA32 (S2A Fig). SETD2 knockdown also did not substantially affect cell number compared to the scramble control 72hr post-transduction with lentivirus particles (S2B Fig). These results suggest that the defect in viral replication observed upon SETD2 knockdown is not due to alterations in cell cycle control. To confirm the importance of SETD2 in productive replication, we utilized suspension in methylcellulose; a commonly used method in the HPV field to induce epithelial differentiation and activate late viral events. Again, we found that transient knockdown of SETD2 using shRNA #2 resulted in reduction in episome copy number in undifferentiated cells (T0) (S3 Fig). Similar to calcium-induced differentiation, SETD2 knockdown resulted in a block in productive viral replication upon differentiation in methylcellulose, without affecting the levels of the differentiation-specific markers involucrin and K10 (S3 Fig). As a final means to examine the importance of SETD2 in viral replication, we utilized a CRISPR/Cas9 genomic editing approach to knockdown SETD2 in CIN612 cells. We designed two single guide RNAs (sgRNA) and used a lentiviral system to establish a heterogenous population of cells exhibiting depleted SETD2, as previously described [61]. These experiments were performed ten days to three weeks after selection when all living cells were puromycin resistant. As shown in Fig 3B, sgRNA #1 and sgRNA #2 resulted in a partial depletion of SETD2 (~70% knockdown) compared to the non-targeting control. Similar to shRNA-mediated knockdown of SETD2, depletion of SETD2 using guide RNAs blocked productive replication upon differentiation (Fig 3B). In addition, sgRNA #2 resulted in a significant decrease (~45%) in episome copy number in undifferentiated cells. Taken together, these data demonstrate that SETD2 is required for productive viral replication and may also contribute to maintenance of episomal copy number in undifferentiated cells.

### H3K36me3 is enriched at the 3’ end of the early region of the HPV31 genome

**Fig 4:**
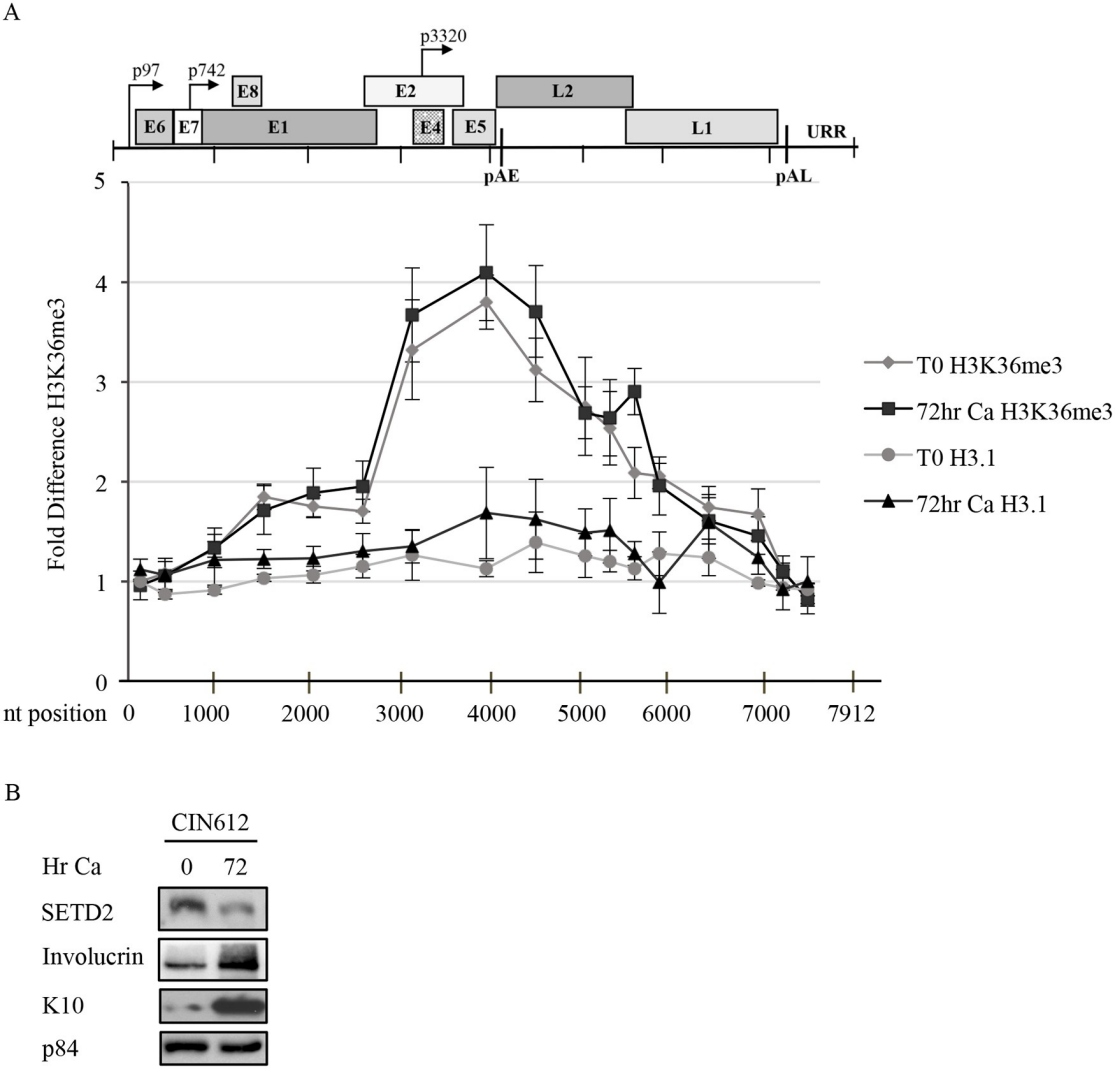
H3K36me3 increases across the early region of the HPV31 genome. (A, B) Chromatin and protein were harvested from CIN612 cells that were undifferentiated (T0) or differentiated in high-calcium medium for 72hr. (A) Chromatin immunoprecipitation (ChIP) was performed using an antibody to H3K36me3 or H3.1 followed by quantitative PCR using 17 primer pairs across the HPV31 genome (S1 Table). To control for differences in viral copy number, PCR data was normalized to input values quantified in parallel for each experiment. The average fold change is graphed and the enrichment is expressed as percent input relative to the first primer set, which is set to one. Averages shown are representative of three independent experiments. Error bars represent means +/- standard error. (B) Western blot analysis was performed using antibodies to SETD2 and to involucrin and K10 as differentiation controls. p84 served as a loading control. Ca = calcium.

**Fig 5:**
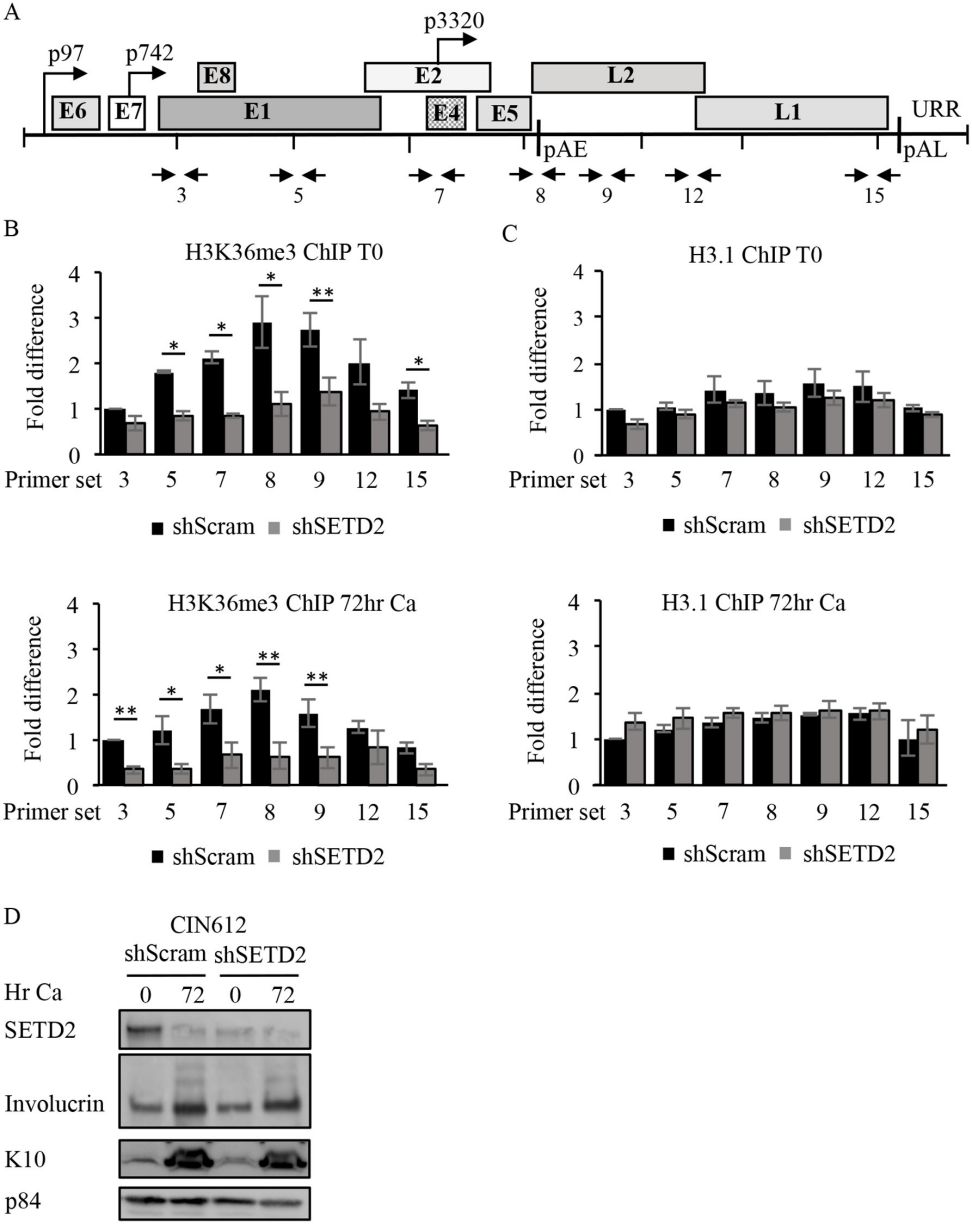
SETD2 is necessary to maintain H3K36me3 on HPV31 chromatin. (A) Schematic of the HPV31 genome showing the location of the primer pairs utilized for ChIP analysis. (B, C) Chromatin and protein were harvested from CIN612 cells transduced with either control shRNA or SETD2 shRNA #2 for 72hr (T0), as well as after differentiation in high calcium medium for 72hr. ChIP was performed using an antibody to (B) H3K36me3 or (C) H3.1 using the indicated primer pairs across the HPV31 genome (primer sequences listed in S1 Table). Data of ChIP signals from three independent experiments were normalized to 1% of input used. Shown in the fold change in H3K36me3 or H3.1 binding relative to the first primer set, which is set to one. Error bars represent means ± standard error. Statistics were assayed using a student’s t test. * p≤ .05 and ** p≤ .01. (D) Western blot analysis was performed using antibodies to SETD2, to involucrin and K10 as differentiation controls and p84 as a loading control. Ca = calcium.

Since SETD2 facilitates cellular processes through recruitment of readers to H3K36me3, we next determined if SETD2 is active on viral chromatin. Chromatin immunoprecipitation (ChIP) for H3K36me3 as well as H3.1 was performed on chromatin harvested from CIN612 cells that were undifferentiated (T0) or differentiated in high calcium medium for 72hr. Using 17 primer pairs across the HPV31 genome (S1 Table), we found by quantitative PCR that H3K36me3 progressively increases across the HPV31 genome, peaking at the 3’ end of the early region, centered over the E2, E4 and E5 open reading frames (ORF) (Fig 4). In contrast, lower levels of the H3K36me3 modification was found in the URR, the E6 and E7 ORFs as well as the early (p97) and late (p742) promoters. Increased H3K36me3 was not due to increased nucleosome density, as H3.1 was fairly constant across the viral genome (Fig 4). Interestingly, the placement of H3K36me3 on viral chromatin was similar in cells undergoing maintenance replication in undifferentiated cells or productive replication upon differentiation. To determine if SETD2 is required for the maintenance of the H3K36me3 mark on the HPV31 genome, we performed ChIP for H3K36me3 as well as H3.1 on chromatin harvested from undifferentiated or differentiated CIN612 cells transiently transduced with either the control shRNA (shScram) or the SETD2 shRNA #2. qPCR was performed using seven primer sets that amplify regions of the HPV31 genome corresponding with either low H3K36me3 (#3, #5, #12, #15) or high H3K36me3 (#7, #8, #9) (Fig 5A, S1 Table). We found that SETD2 knockdown resulted in a significant decrease in H3K36me3 across the HPV31 genome in undifferentiated as well as differentiated cells at almost every region examined (Fig 5B). In contrast, SETD2 knockdown had minimal effect on levels H3.1 (Fig 5C). The results indicate H3K36me3 enrichment on viral chromatin requires SETD2 activity.

### H3K36me3 is required for productive viral replication

**Fig 6:**
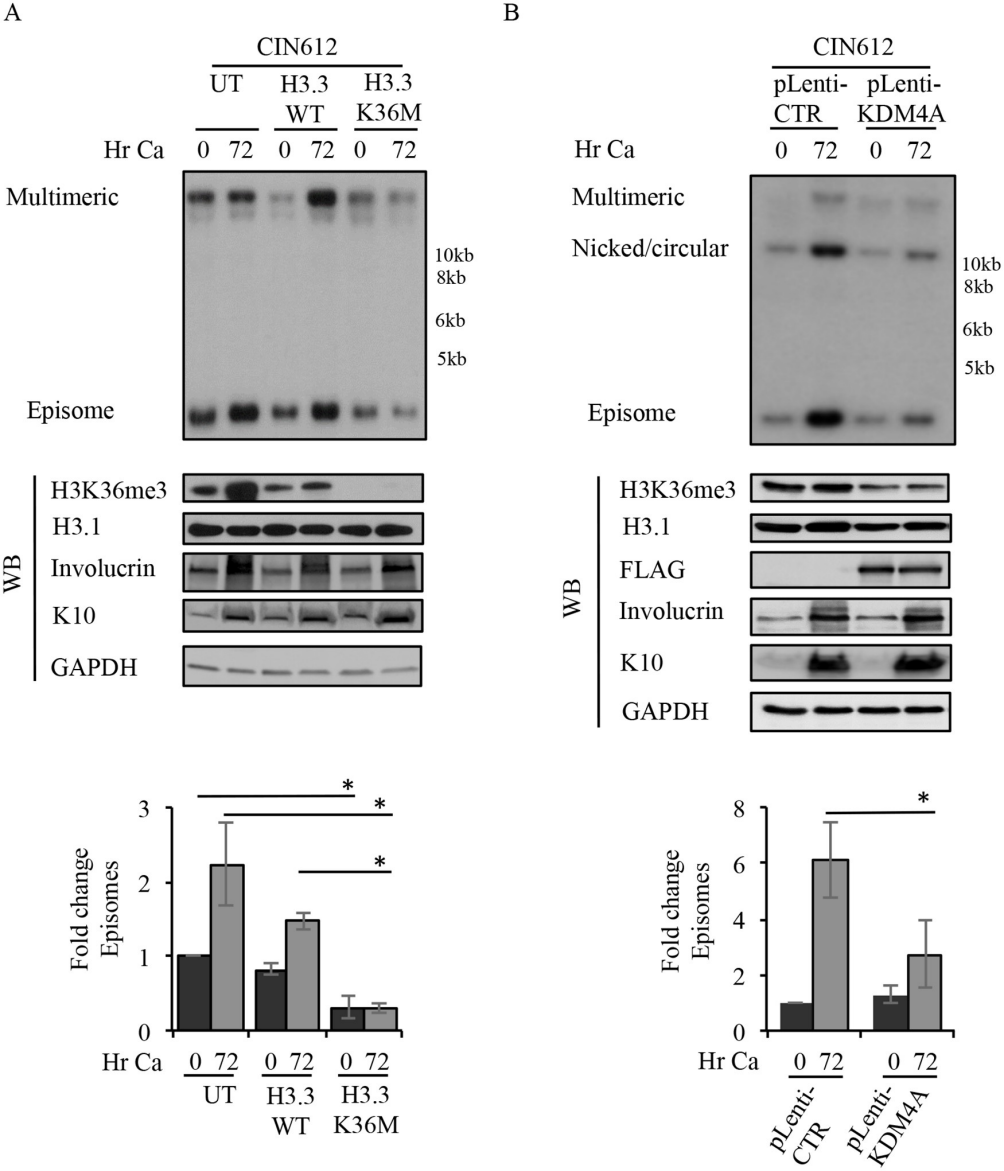
H3K36me3 is required for productive viral replication. (A) CIN612 cells were left untreated (UT) or transduced with lentivirus expressing either wild-type (WT) H3.3 or the H3.3K36M mutant. 72hr post-transduction, cells were harvested as a T0 (undifferentiated) or differentiated in high calcium medium for 72hr. (B) CIN612 cells were transduced with either pLenti-control (CTR) or pLenti-FLAG-KDM4A. Following selection in puromycin, cells were harvested as a T0 (undifferentiated) or were induced in high calcium for 72hr. For (A) and (B) DNA and protein were harvested at the indicated time points. DNA was digested with BamHI (non-cutter) and Southern blotting analysis was performed to analyze episome copy number using the HPV31 genome as a probe. Western blot analysis was performed to examine the levels of H3K36me3, with H3.1 serving as a loading control. Involucrin and K10 were used as markers of differentiation and GAPDH as loading control. For (B) western blot analysis was performed using an antibody to FLAG to detect KDM4A. For (A) and (B), fold change in episome copy number was determined by performing densitometry of episomal bands from three independent experiments using ImageJ software. Graphed is the average fold change relative to (A) UT T0 and (B) pLenti-CTR, which are set to one. Error bars represent means ± standard errors. Statistics were assayed using a student’s t test. * p < 0.05. Ca = calcium.

Our finding that SETD2 is necessary for HPV31 replication suggests that the H3K36me3 modification may also be required. To examine this, we utilized two methods to reduce H3K36me3 in a SETD2-independent manner: (1) expression of a dominant negative H3.3K36 containing a K36M (lysine to methionine) mutation (H3.3K36M) and (2) expression of the KDM4A demethylase. H3.3K36M cannot be trimethylated but results in a global loss of H3K36me3 without affecting other histone methylations [62]. KDM4A specifically removes the trimethyl mark from H3K36 as well as H3K9 [63]. CIN612 cells were transduced with lentivirus to express either wild-type H3.3 or the mutant H3.3K36M transgene. After 72hr, CIN612 cells were harvested as a T0 (undifferentiated) or differentiated in high calcium medium for 72hr. As previously shown, expression of the H3.3K36M mutant resulted in a global decrease in H3K36me3 compared to the wild-type H3.3 control (Fig 6A) [62]. Similar to knockdown of SETD2, we found that expression of H3.3K36M resulted in approximately a 70% reduction in episomal copies in undifferentiated cells and blocked productive viral replication upon differentiation, while cells expressing the wild-type H3.3 exhibited a replication phenotype similar to untreated (UT) cells, with a moderate effect on productive replication (Fig 6A). To examine the effect of KDM4A overexpression on viral replication, CIN612 cells were stably transduced with either lentivirus harboring empty vector or FLAG-KDM4A. Cells were harvested either prior to or after inducing differentiation in high calcium medium for 72hr. As shown in Fig 6B, CIN612 cells expressing FLAG-KDM4A had lower levels of H3K36me3 compared to the control, as previously demonstrated [53, 63]. In addition, KDM4A overexpression resulted in a defect in viral genome amplification upon differentiation (Fig 6B). Collectively, our studies using SETD2 depletion, expression of the H3.3K36M mutant as well as KDM4A, which share the common feature of reducing H3K36me3, indicate that H3K36me3 is required for viral replication. Furthermore, given that H3K36me3 is enriched on the HPV31 genome, our data suggest that SETD2 may regulate viral processes through the recruitment of cellular readers to the H3K36me3 mark.

### SETD2 activity influences viral RNA processing

**Fig 7:**
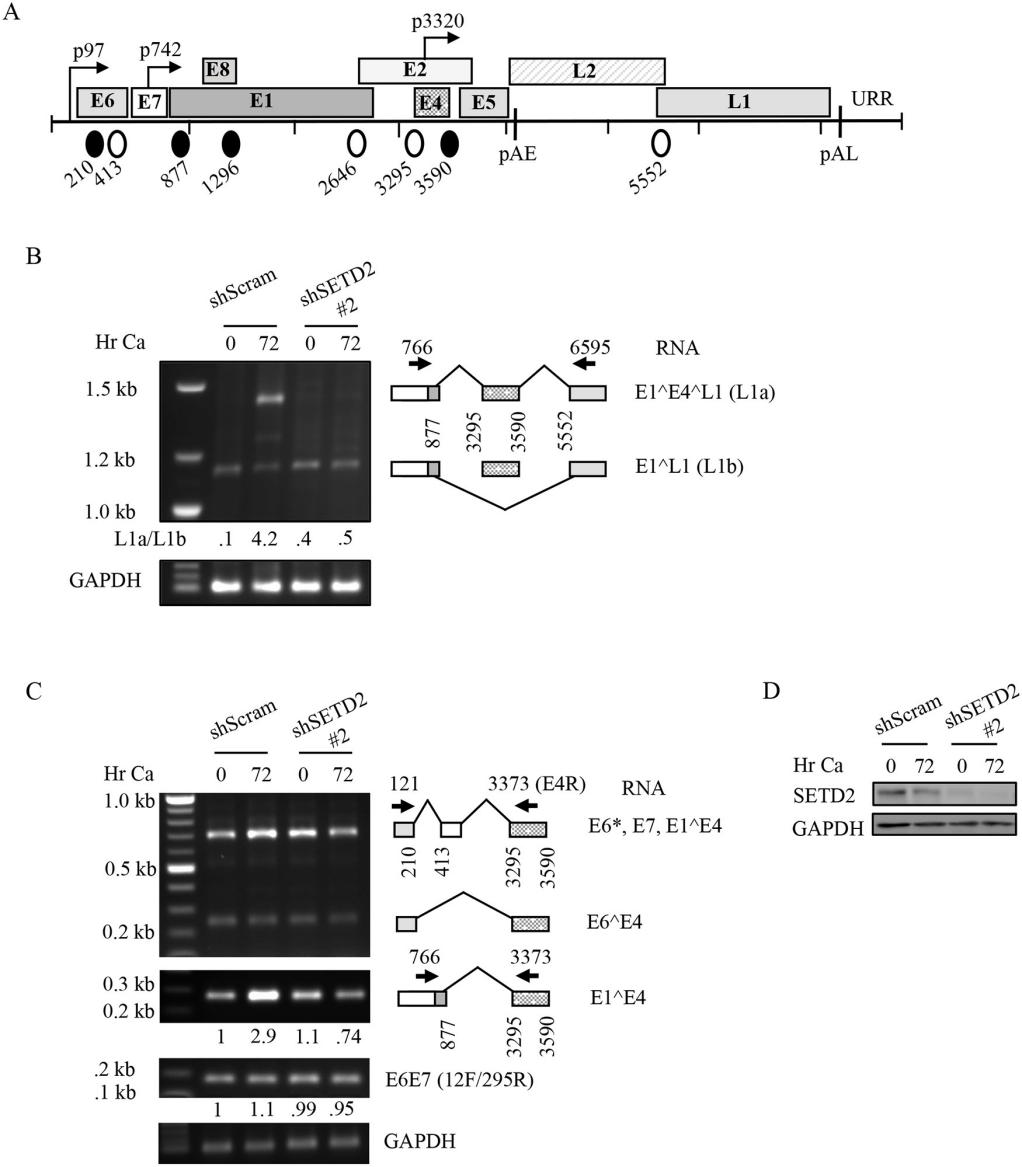
SETD2 knockdown affects processing of late viral RNAs. (A) Schematic of the HPV31 genome showing 5’ splice donor (SD) (closed circles) and 3’ splice acceptor (SA) (open circles) sites. Open reading frames are indicated by the shaded boxes, and promoters are indicated by the arrows. The early (pAE) and late (pAL) polyadenylation sites are indicated at the end of the E5 and L1 ORFs, respectively. (B) CIN612 cells were transiently transduced with lentivirus expressing either a control shRNA (shScram) or SETD2 shRNA #2. 72hr post-transduction, cells were harvested as a T0 (undifferentiated) or differentiated in high calcium medium for 72hr. At each time point, RNA and protein were harvested. (B) End-point PCR amplification was performed (35 cycles) using a 5’ primer in the E7 ORF (nt 766) and a 3’ primer in the L1 ORF (nt 6595), as well as primers specific to GAPDH. Primer sequences are listed in S1 Table. PCR products were gel purified and sequenced. Shown is a graphical representation of the identified products. Relative levels of L1a and L1b were determined by densitometry using ImageJ software. Values shown represent the ratios of L1a/L1b at each time point. (C) PCR amplification was performed for 25 cycles using the indicated primers to the early region as well as primers specific to GAPDH. Primer sequences are listed in S1 Table. PCR products generated using the 121F/3373R (E4R) primer pair were gel purified and sequenced. Shown is a graphical representation of the identified products. Relative levels of E1^E4 and E6E7 were determined by densitometry using ImageJ software and normalized to GAPDH. Shown is the fold difference relative to shScram T0, which is set to 1. (D) Western blot analysis was performed using antibodies specific to SETD2 and GAPDH. Shown are representative images of three independent experiments. Ca = calcium. WB = western blot.

Transcription of HPV31 mRNAs is regulated by the early promoter (p97) in undifferentiated cells and by the late promoter (p742) upon differentiation (Fig 7A). HPV genes are transcribed as polycistronic transcripts that are alternatively spliced to yield individual gene products [56]. Alternative splicing is thus a key control mechanism of HPV gene expression and is accomplished through multiple splice donor and splice acceptor sites (Fig 7A). SETD2-mediated H3K36me3 provides a docking site for chromatin adapter proteins (e.g. MRG15, p52) that in turn recruit splicing factors to drive splice site selection [49, 52]. We have found that H3K36me3 is enriched over the most commonly used 3’ splice acceptor at 3295 (SA3295), located at the 5’ end of the E4 exon, the splice donor at 3590 (SD3590), located at the 3’ end of the E4 exon, the putative p3320 promoter located in the E4 ORF as well as the early polyadenylation site located downstream of the E5 ORF. Use of SA3295 produces transcripts encoding E6, E7, E4 and E5 as well as the capsid proteins L1 and L2 [6, 19, 20, 64], while SD3590 is utilized for the production of L1 RNAs upon differentiation [19, 20]. To determine if SETD2 activity is important for viral splicing, we extracted RNA from undifferentiated or differentiated CIN612 cells transiently transduced with either control shRNA or SETD2 shRNA #2. We first determined if splicing in the late region is affected by SETD2 knockdown. To ensure detection of low-level transcripts, we performed end-point PCR (35 cycles) using a 5’ primer that anneals at nucleotide 766 in the E7 open reading frame (E7F) that is upstream of the splice donor site SD877, and a 3’ primer that anneals at nucleotide 6595 in the L1 open reading frame (L1R), downstream of SA3295 and SA5552. As shown in Fig 7B, amplification of RNA from CIN612 cells containing the control shRNA resulted in two major products upon differentiation, one of which was also present in undifferentiated cells. Some minor products were also detected. The two major splice products of 1.5kb and 1.2kb were identified by sequencing and shown to be two alternatively spliced L1 RNAs, spliced at 877^3295 and 3590^5552 (E1^E4^L1 RNA) (L1a) and 877^5552 (E1^L1 RNA) (L1b), respectively. Upon SETD2 knockdown, levels of the E1^E4^L1 RNA (L1a) were substantially reduced (Fig 7B). Furthermore, we found that the ratio of the two L1 splice variants (L1a/L1b) was altered upon SETD2 knockdown in differentiating cells, suggesting that the H3K36me3 modification is necessary for efficient expression of the E1^E4^L1 mRNA. A similar change in the L1 splice variant ratio was observed using semi-quantitative RT-PCR (S4A Fig). Furthermore, using the E4F/L1R primer pair to PCR amplify across the E4^L1 splice junction (3590^5552), we found that SETD2 knockdown resulted in a substantial decrease in generation of this spliced product (S4A Fig), suggesting that the presence of the H3K36me3 mark over SD3590 may be required for efficient use of this splice site upon differentiation.

To determine if splicing to SA3295 is also affected by SETD2 knockdown, we first utilized a 5’ primer that anneals at nucleotide (nt) 121 (121F), upstream of the first splice donor (SD) site (nt 210), and a 3’ primer that anneals at nt 3452 (E4R) that is downstream of the four splice acceptor (SA) sites in the early region of HPV31. Amplification of RNA from CIN612 cells containing either the control or SETD2 shRNA #2 resulted in two major products in undifferentiated and differentiated cells (Fig 7C). The two splice products of 0.7kb and 0.25kb were identified by sequencing and shown to be spliced at 210^413 and 877^3295 (E6*, E7, E1^E4 RNA) and 210^3295 (E6^E4 RNA), respectively. As shown in Fig 7C, both splice products were readily detected upon SETD2 knockdown in undifferentiated cells, with a slight decrease observed upon differentiation, suggesting that splicing to SA3295 is not severely compromised. To include transcripts produced from the late promoter (p742), which is located in the E7 ORF, we utilized the E7F primer (nt 766) and a reverse primer located in the E4 exon (E4R, nt 3452) to amplify across the E1^E4 splice junction (877^3295). In contrast to splicing across the E4^L1 junction, we found that there was not a defect in splicing across the E1^E4 junction upon SETD2 knockdown, with spliced E1^E4 RNAs detected in undifferentiated and differentiated cells (Fig 7C). Similarly, using a 5’ primer that anneals at nt 1270 and the 3’ E4R primer, we found that splicing still occurred across the 1296^3295 junction to generate the E8^E2C RNA upon SETD2 depletion (S4A Fig). In addition, using the E7F and E2R (nt 2807) primer pair we observed minimal effect of SETD2 knockdown on splicing across the 877^2646 junction to generate the spliced E2 product in either undifferentiated or differentiated cells (S4B Fig). While SETD2 depletion did prevent the differentiation-dependent increase in levels of spliced E1^E4, E8^E2C and E2, this could stem from the inability of HPV to productively replicate in the absence of SETD2 activity, resulting in fewer templates for transcription. The decrease in E1^E4^L1 transcripts also likely contributes to the reduction in E1^E4 observed upon SETD2 knockdown upon differentiation. Not surprisingly, the levels of E5, which is present on polycistronic transcripts containing E4, were also lower upon differentiation in SETD2 knockdown cells compared to scramble control (S4B Fig). Further characterization of splicing in the early region revealed minimal impact of SETD2 knockdown on the relative levels of unspliced E6E7 transcripts produced from the early promoter p97 using the 121F/295R primer pair (Fig 7C). Overall, these results indicate that SETD2-mediated H3K36me3 on viral chromatin does not markedly affect splicing in the early region, but does influence splice site selection for the production of late L1 RNAs, which may occur through the recruitment of splicing factors.

### ATM activation is necessary for H3K36me3 maintenance on viral chromatin

**Fig 8:**
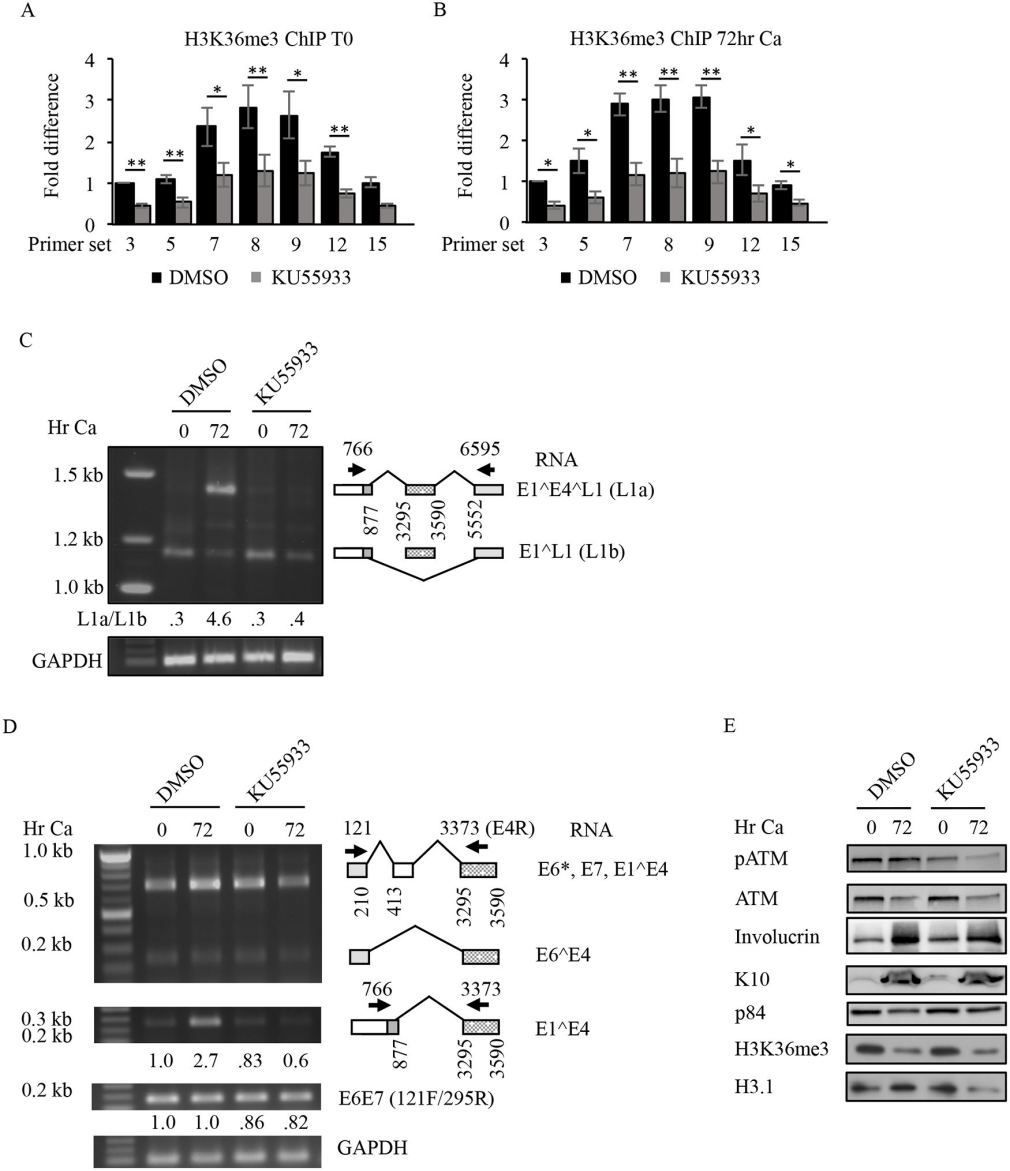
Inhibition of ATM kinase activity decreases H3K36me3 on the HPV31 genome and alters processing of late viral RNAs. Chromatin was harvested from (A) undifferentiated CIN612 cells treated with DMSO or 10uM of the ATM inhibitor KU55933 for 24hr, and from (B) CIN612 cells differentiated in high calcium medium for 72hr in the presence of DMSO or 10uM KU55933. ChIP was performed using an antibody to H3K36me3 using primer pairs indicated in Fig 4A (S1 Table). Data of ChIP signals from three independent experiments were normalized to 1% of input used. Shown in the fold change in H3K36me3 relative to the first primer set, which is set to one. Error bars represent means ± standard error. Statistics were assayed using a student’s t test. * p≤ .05 and ** p≤ .01. (C, D) RNA and (E) protein were harvested from undifferentiated CIN612 cells (T0) treated with DMSO or 10uM KU55933 as well as from CIN612 cells differentiated in high calcium medium (72hr) in the presence of DMSO or 10uM KU55933. (C) Following cDNA synthesis, end-point PCR amplification was performed (35 cycles) using a 5’ primer in the E7 ORF (nt 766) and a 3’ primer in the L1 ORF (nt 6595), as well as primers specific to GAPDH (S1 Table). PCR products were gel purified and sequenced. Shown is a graphical representation of the identified products. Relative levels of L1a and L1b were determined by densitometry using ImageJ software. Values shown represent the ratio of L1a/L1b at each time point. (D) PCR amplification was performed (25 cycles) using the indicated primers to the early region as well as primers specific to GAPDH. The PCR products generated using the 121F/3373R (E4R) primer pair were gel purified and sequenced. Shown is a graphical representation of the identified products. Relative levels of E1^E4 and unspliced E6E7 were determined by densitometry using ImageJ software and normalized to GAPDH. Shown is the fold difference relative to DMSO T0, which is set to 1. Primer sequences are listed in S1 Table. (E) Western blot analysis was performed using antibodies to phosphorylated ATM on serine 1981 (pATM), total ATM and H3K36me3. Involucrin and K10 were used as differentiation controls. p84 and H3.1 served as loading controls. Ca = calcium.

ATM has been shown to inhibit the demethylase KDM2A, which removes di-methyl groups from H3K36, and also negatively impacts KDM4A levels through proteasome-dependent degradation [65, 66]. Interestingly, a recent study using integrated subgenomic HPV16 reporters demonstrated that exogenously-induced DNA damage leads to production of spliced late L1 mRNAs, specifically the E1^E4^L1RNA, in an ATM-dependent manner [67]. ATM activation is required for productive replication of HPV31 [27], though whether ATM contributes to the viral life cycle through epigenetic modifications on viral chromatin is unknown. To determine if ATM activity is required for maintenance of H3K36me3 on viral chromatin, we performed ChIP on chromatin harvested from undifferentiated (T0) and differentiated (72hr Ca) CIN612 cells that were treated with DMSO or 10uM of the ATM inhibitor KU55933 (Fig 8A). qPCR was performed using primer pairs to areas of the HPV31 genome exhibiting high and low levels of H3K36me3. The location of the primers is shown in Fig 5A and the sequences are listed in S1 Table. Similar to SETD2 knockdown, we found that ATM inhibition results in a significant decrease in H3K36me3 across most regions of the HPV31 genome (Fig 8A and 8B), without affecting levels of H3.1 (S5 Fig). Using the same primer pairs described above, we found that ATM inhibition, similar to that of SETD2 knockdown, resulted in decreased levels of the E1^E4^L1 RNA (L1a) upon differentiation and altered the ratio of L1a to L1b (Fig 8C and S6A Fig). Furthermore, inhibition of ATM activity resulted in decreased splicing across the E4^L1 junction upon differentiation (S6A Fig). In contrast, splicing in the early region was minimally affected, including the generation of the E6*, E7, E1^E4 and E6^E4 RNAs (Fig 8D), splicing across the 877^3295 junction (E1^E4) (Fig 8D) and the 1296^3295 junction (E8^E2C) (S6A Fig) as well as generation of the spliced E2 product (877^2646) (S6B Fig). In addition, the relative levels of unspliced E6E7 transcripts from p97 were minimally affected by ATM inhibition (Fid 8D). While ATM inhibition did result in decreased levels of E1^E4, E8^E2C, and E5 upon differentiation compared to the DMSO control, this again likely reflects the defect in productive viral replication. The decreased levels of E1^E4 may also be due to the defect in generating the E1^E4^L1 spliced product. Interestingly, in contrast to SETD2 knockdown, we found that inhibition of ATM activity did not result in a global loss of H3K36me3 as detected by western blot analysis (Fig 8E), suggesting that ATM’s affects on H3K36me3 may be largely restricted to viral chromatin. Importantly, these studies indicate that ATM activation is important for maintenance of H3K36me3 on viral chromatin and may regulate viral processes through the recruitment of H3K36me3 readers.

## Discussion

In this study, we demonstrate that the HPV life cycle is epigenetically regulated by a cellular methyltransferase and the H3K36me3 mark. Our data demonstrate that SETD2 is active on HPV31 DNA and that placement of the H3K36me3 mark on viral chromatin is necessary to support viral replication and promote processing of late viral RNAs. In addition, our studies identify a novel role for the ATM DNA damage kinase in the epigenetic regulation of viral processes by regulating the levels of H3K36me3 on viral DNA. Furthermore, we have found that E7 increases SETD2 levels post-transcriptionally in a manner dependent on its Rb binding domain. These studies identify a mechanism by which HPV manipulates epigenetic pathways for viral processes and also reveal an important role for E7 in the epigenetic regulation of the viral life cycle.

The distribution of H3K36me3 on the HPV31 genome resembles that of a cellular gene body: progressively increasing across the early region and peaking at the 3’ end. A previous study using subgenomic HPV16 reporter plasmids reported a similar increase in H3K36me3 over the early polyadenylation site (pAE), located just downstream of E5 [68]. This result, coupled with our finding that HPV16 positive cells have increased levels of SETD2, suggests that regulation of viral processes by SETD2 through H3K36me3 may extend to other HPV types. H3K36me3 is associated with active transcription, and the distribution of H3K36me3 could potentially result from transcription from the early promoter (p97) and the generation of transcripts polyadenylated at the pAE [64]. However, upon differentiation we did not observe a similar increase in H3K36me3 over the late region when the late promoter is active and transcripts are polyadenylated at the late site (pAL), located downstream of L1. These results suggest that increased transcription through the late region is insufficient to increase H3K36me3. However, several studies have shown that splicing is a determinant of H3K36me3 placement, with formation of the spliceosome enhancing the recruitment of SETD2 to RNA pol II [69, 70]. As mentioned, the H3K36me3 peak encompasses the most commonly used 3’ splice site on the HPV genome (SA3295 for HPV31) located at the 5’ end of the E4 ORF, which is important for the generation of transcripts encoding E6, E7, E4 and E5 as well as the capsid proteins L1 and L2 [11, 19, 20]. The frequent use of SA3295 may increase the recruitment of SETD2 to RNA pol II leading to enhanced placement of H3K36me3 at the end of the early region in both undifferentiated and differentiated cells. In addition, H3K36me3 is enriched over the 5’ splice site SD3590, which is located at the 3’ end of E4 and is activated upon differentiation to allow for expression of L1 [64]. These results suggest that splicing, rather than transcription, may be the major driver in H3K36me3 placement on HPV chromatin.

While splicing may affect the placement of H3K36me3 on the HPV genome, our results indicate that H3K36me3 is important for splice site selection on late viral RNAs. Depletion of SETD2 as well as inhibition of ATM kinase activity reduced H3K36me3 on the viral genome and resulted in exclusion of the E4 ORF from a late E1^E4^L1 mRNA upon differentiation. While SETD2 knockdown and ATM inhibition had limited effect on the use of the 3’ splice site SA3295, we found that splicing from SD3950 at the 3’ end of E4 to SA5552 at the 5’ end of L1 (E4^L1) was remarkably reduced. Furthermore, the ratio of E1^E4^L1 to E1^L1 was substantially altered by SETD2 knockdown and ATM inhibition, indicating that H3K36me3 on viral DNA influences splice site selection to ensure efficient E4^L1 splicing upon differentiation. H3K36me3 facilitates splice site selection by creating a docking site for the chromatin adapter proteins MRG15 and Psip1/p52, which in turn recruit splicing factors [49, 52]. MRG15 recruits PTB (polypyrimidine tract binding protein) to alternatively spliced exons, while Psip1/p52 recruits SRSF1, a member of the splicing enhancing serine-arginine rich (SR) protein family. Interestingly, both PTB and SRSF1 have been reported to play a role in the regulation of HPV splicing [56]. In addition, SRSF3, which also binds Psip1/p52, has recently been shown to regulate expression of HPV31 and HPV16 L1 mRNAs, specifically expression of the E4^L1 mRNA [49, 71]. Furthermore, PTB has been shown to play a role in the expression of L1 by relieving suppression of the late splice sites SD3950 [72]. PTB may be recruited to H3K36me3 on viral chromatin through MRG15 specifically during the late stages of the viral life cycle to promote L1 expression. Future studies will determine if SRSF1, SRSF3 and PTB recruitment occurs in an H3K36me3-dependent manner.

ATM has been recently shown to affect the splicing of late mRNAs expressed from integrated HPV16 subgenomic reporter plasmids, which is postulated to occur through phosphorylation of BRCA1 and the recruitment of splicing factors to viral RNAs [67]. Our studies suggest that ATM activity regulates splicing of late L1 RNAs expressed from HPV31 episomes through the maintenance of H3K36me3 on viral chromatin. Whether this also involves BRCA1 is currently unknown, though we have previously shown that BRCA1 is required for productive viral replication [73]. The mechanism by which ATM regulates H3K36me3 on viral chromatin is currently unclear. In response to DNA damage, ATM inhibits the activity of the KDM2A demethylase, which removes dimethyl groups from H3K36 and could in turn prevent trimethylation by SETD2. Intriguingly, ATM activity has also been shown to promote the proteasome-dependent degradation of KDM4A [65], which we have found blocks productive replication upon overexpression. KDM4A stability is regulated by the RNF8 and RNF168 ubiquitin ligases, which are recruited to sites of DNA damage in an ATM-dependent manner [65]. While multiple effectors of ATM have been shown to localize to sites of HPV replication [23], whether RNF8 and RN168 are also recruited to HPV genomes has not been examined. The regulation of H3K36me3 by ATM will be a focus of future investigation.

In this study, we have found that H3K36me3 depletion by SETD2 knockdown, expression of the H3.3K36M mutant or the KDM4A demethylase blocks productive viral replication. Importantly, previous studies have shown that SETD2 knockdown, as well as H3.3K36M and KDM4A expression do not affect cell cycle progression [53], and we have found similar results, indicating that the effect of H3K36me3 deficiency on viral replication is not due to an inability to remain active in the cell cycle. How SETD2 contributes to HPV31 replication is unclear, but could occur through the recruitment of several readers of the H3K36me3 mark [74]. SETD2 promotes nucleosome reassembly behind RNA pol II through recruitment of the FACT (Facilitates Chromatin Transcription) complex to H3K36me3 [50]. Loss of H3K36me3 leads to reduced FACT loading and a decrease in nucleosome density, which could impact viral replication as well as transcription. In addition, previous studies from our lab and others demonstrated that DNA repair processes regulated by SETD2-mediated H3K36me3 are required for HPV replication [23]. SETD2 promotes error-free homologous recombination (HR) repair within transcriptionally active regions in response to double-strand DNA breaks (DSB) as well as replication stress through the constitutive binding of LEDGF and PALB2, respectively, to H3K36me3 [53, 55]. In response to DSBs, LEDGF allows for the recruitment of the CtIP nuclease, resulting in DSB resection and formation of single-strand DNA that is bound by the HR recombinase Rad51 [53]. PALB2 associates with H3K36me3 through MRG15 and facilitates swift linkage of the HR repair factors BRCA2 and BRCA1, which recruit Rad51 to protect and/or repair nearby replication forks suffering from replication stress [55]. In addition to BRCA1, we have found that Rad51 is also required for productive viral replication [57]. Rad51 binds to HPV31 genomes, but whether this occurs in an H3K36me3-dependent manner and protects viral genomes from DNA damage and replication stress is unclear. ATM activation promotes HR repair and is required for productive viral replication [27, 75]. Future studies will determine if SETD2 and ATM-dependent regulation of H3K36me3 on viral chromatin ensures the recruitment of HR factors to viral genomes.

Our studies indicate that E7 increases SETD2 levels post-transcriptionally through an increase in protein half-life, in a process that requires E7’s Rb binding domain. How E7 increases the SETD2 protein half-life is currently unknown. SETD2 stability is regulated by the ubiquitin ligase SPOP, which forms an ubiquitin E3 ligase complex with cullin 3 (CUL3) and ring-box 1 (ROC1/RBX1) [60]. E7 proteins bind CUL3 and may in turn block formation of the SPOP/CUL3 complex [76]. SETD2 contains a conserved C-terminal SRI domain for interaction with RNA pol II and a SET domain responsible for catalyzing substrate methylation [77]. Mutations in the SET domain of SETD2 and the conserved SRI domain of yeast Set2 have been shown to influence protein stability through loss of binding to histone H3 and RNA pol II, respectively [78, 79]. E7 significantly affects host gene expression and may protect SETD2 from degradation by promoting transcription and interaction with RNA pol II or histone H3 [80]. Interestingly, recent studies have shown that SPOP and SETD2 are targeted for degradation by the APC/C^cdh1^ complex in G1 [81, 82]. HPV16 E7 interferes with the degradation of APC/C^cdh1^ substrates, though whether this occurs in a manner dependent on the Rb binding domain is unclear [83]. E7 may therefore have multiple mechanisms to counteract SETD2 protein degradation.

SETD2 is typically associated with tumor suppressor activity and is often mutated in several cancer types [77]. In contrast, we have found that high-risk HPV positive cells exhibit increased SETD2 levels that are necessary for viral replication and viral RNA processing. Our studies suggest that the E7-mediated increase in SETD2 plays a role in regulating viral processes through placement of the H3K36me3 mark on viral chromatin. Understanding how SETD2 regulates the viral life cycle through binding of alternative H3K36me3 effector proteins to viral chromatin will be a focus of future investigation. However, an equally important area will be to understand the impact of increased SETD2 levels on the cellular landscape and how these epigenetic alterations may promote viral persistence, a major risk factor for the development of cancer. Our results provide further support that the effects of E7 and E6 on host epigenetic modifiers have consequences on viral chromatin and viral processes. A more complete understanding of how HPV manipulates epigenetic pathways to facilitate the viral life cycle will provide further insight into how these pathways can be exploited for the treatment of HPV-associated diseases.

## Materials and methods

### Cell culture

Human foreskin keratinocytes (HFKs) were isolated from neonatal foreskin tissue and were maintained in Dermalife keratinocyte growth medium (KGM; Lifeline Cell Technology), as described previously. [84]. HFKs containing wild-type HPV31, HFK-31 ^Δ^LHYCE and HFK-16 were generated by co-transfecting HFKs with re-circularized HPV31 (or ^Δ^LHYCE) genome (excised from pBR-322min) or HPV16 (excised from p1203 PML2d) along with a pSV2-neo resistance plasmid using PolyJet transfection reagent (Signagen Laboratories), followed by eight days of selection in G418 (Sigma), as described [59]. E7-expressing HFKs were made using pLXSN encoding wild-type HPV31 E7 and the E7 ^Δ^LHCYE mutant, along with G418 (Sigma) selection, as described [41]. surviving populations were pooled and expanded for analysis. All lines were cultured in E medium supplemented with 5 ng/ml mouse epidermal growth factor (EGF; BD Biosciences) in the presence of mitomycin C-treated NIH J2 3T3 murine fibroblast feeder cells (obtained from Lou Laimins, Northwestern University) [85]. When necessary, J2 feeders were removed from HPV-positive cells by incubation with 1 mM EDTA in phosphate-buffered saline (PBS). Human embryonic kidney 293T cells and NIH 3T3-derived PT67 cells were obtained from Lou Laimins (Northwestern University) and cultured in Dulbecco’s modified Eagle’s medium (DMEM; Life Technologies) supplemented with 10% bovine growth serum (BGS; ThermoFisher Scientific). Calcium-induced differentiation was performed as previously described [27]. Where indicated, cells were treated with 10uM of the ATM kinase inhibitor KU55933 (Tocris Bioscience) or DMSO for the designated amount of time.

### Ethics statement

Human keratinocytes were isolated from discarded, de-identified foreskins obtained from routine circumcisions performed at UNC hospital in Chapel Hill, NC. Because these tissues were anonymous (therefore not requiring consent), not collected specifically for our research, and would have been discarded otherwise, the Office of Human Research Ethics at UNC-Chapel Hill has determined that our use of human foreskin keratinocytes does not constitute human subjects research as defined under federal regulations [45 CFR 46.102 (d or f) and 21 CFR 56.102(c)(e)(l)] and does not require IRB approval.Study number 18–0950.

### Differentiation in methylcellulose

CIN612 cells were suspended in 1.5% methylcellulose as previously described [86]. Cells were harvested as an undifferentiated sample (T0) or after 48hr differentiation in methylcellulose. At each time point, cells were harvested for DNA and protein as described below.

### Plasmids

pBR322min containing the HPV31 and–HPV31 ^Δ^LHCYE genome has been described [87]. p1203 PML2D containing HPV16 was obtained from Addgene. pLXSN-HPV31 E7, pLXSN-HPV31 E6/E7 and pLXSN-HPV31 ^Δ^LHCYE plasmids were described previously [59]. The KDM4A ORF was cloned into pLenti-C-Myc-DDK-IRES-Puro (Origene) within the SgfI/MluI cloning site using T4 DNA ligase (Invitrogen). The H3.3 and H3.3K36M lentiviral plasmids were kind gifts from Dr. David Allis and were previously described [62].

### Lentivirus production and transduction

Pre-validated lentiviral shRNAs specific to SETD2 were purchased from Sigma Aldrich (#1 TRCN0000237839, #2 TRCN0000237837). A scramble control shRNA cloned into the pLKO.1-puro background was obtained from the UNC Lentiviral Core Facility (Chapel Hill, NC). Lentivirus particles expressing control or SETD2 shRNAs, FLAG-KDM4A, H3.3 or H3.3K36M were prepared as previously described [88]. Each plasmid was transiently transfected into 293T cells, along with Gag-Pol-Tet-Rev plasmid DNA and vesicular stomatitis virus G (VSV-G) plasmid DNA using polyethyleneimine (PEI) (VWR). Supernatants containing lentivirus were harvested 72 hours post-transfection. CIN612 cells were transduced with 5ml viral supernatant in the presence of 4 ug/ml Polybrene (Sigma). The medium was changed 24hr post-transduction and the cells were allowed to grow for another 48hr. At this point, cells were harvested or differentiated, or stably selected in puromycin.

### SETD2 depletion by CRISPR/Cas genomic editing

Two different sgRNAs targeting SETD2 were designed using CRISPRdirect (https://crispr.dbcls.jp/) and cloned into pLentiCRISPRv2 (kind gift from Dr. Gaurov Gupta), as described previously [89]. Lentivirus particles expressing each sgRNA or vector control were generated as previously described [88]. CIN612 cells were transduced with 5ml viral supernatant in the presence of 4 ug/ml Polybrene (Sigma). 48hr post-transduction, cells were selected in medium containing 5 μg/ml of puromycin for six days. The target guide sequences were sgRNA #1 ACTCTGATCGTCGCTACCATAGG and sgRNA #2 GAGAGAGGACGCGCTATTCTCGG. The primers utilized to generate the SETD2 sgRNAs are listed in the S1 Table. SETD2 depletion was confirmed by western blotting.

### Western blot analysis

Lysate harvesting and western blot analysis was performed as previously described [57]. To quantify the levels of keratin 10 (K10), insoluble cell pellets obtained from RIPA-SDS lysis were resuspended in 8 M urea, 10% β-mercaptoethanol, 2 mM PMSF, and incubated at room temperature while rotating for 30 min, as described previously [90]. The insoluble debris was removed by centrifugation at 14,000 rpm at 4’C. Primary antibodies used: anti-SETD2 (Kind gift from Dr. Brian Strahl, Epicypher), anti-H3K36me3, anti-cyclin B, anti-CDK2 (Abcam), anti-H3.1 (Active Motif), p84 (GeneTex), anti-Involucrin, anti-keratin 10, anti-CDC25C, anti-cyclin A, anti-cyclin E and anti-GAPDH (Santa Cruz), anti-CDK1, anti-RPA32 (Bethyl laboratories) and anti-Flag (Sigma). Secondary antibodies used were horseradish peroxidase (HRP)-conjugated anti-rabbit (Cell Signaling Technology) and HRP-conjugated anti-mouse (GE Life Sciences). Western blots were developed using Clarity Western ECL blotting substrate (Bio-Rad). Images were captured on either autoradiography film or Biorad ChemidocMP imaging system. Blots were analyzed with Biorad Imagelab 5.0 software.

### Southern blot analysis

DNA isolation and Southern blotting were performed as previously described [91]. Briefly, cells were harvested in DNA lysis buffer (400mM NaCl, 10mM Tis pH 7.5 and 10mM EDTA), then lysed by the addition of 30uL 20% SDS. Samples were subsequently treated with 15ul of 10mg/mL proteinase K overnight at 37°C. DNA was extracted using phenol chloroform, followed by ethanol precipitation in the presence of sodium acetate. 5ug of DNA was digested with either BamHI (New England Biolabs) (does not cut the genome), or HindIII (New England Biolabs) (cuts the genome once). DNAs were resolved on a 0.8% agarose gel for 15 h at 40 V and were then transferred to a positively charged nylon membrane (Immobilon-Ny+; EMD Millipore). The DNA was fixed to the membrane via UV irradiation and then hybridized to a radioactive DNA probe consisting of ^32^P-labeled linearized HPV31 genome.

### Measurement of protein half-life

HFKs, HFK-31, HFK-31 ^Δ^LHCYE and CIN612 cells were grown in 10cm dishes until approximately 80% confluent. Cells were treated with 50 ug/ml cycloheximide and whole cell lysates were harvested at the indicated time points. Western blot analysis was performed using 50ug of total protein as described previously [25]. Westerns were digitally imaged using the Bio-Rad Chemidoc MP system, and densitometry was performed with the Biorad ImageLab 5.0 software.

### RNA extraction

Total RNA was extracted using RNA Stat 60 (Tel-Test) followed by DNase digestion (Promega) and was reverse transcribed using the Superscript Vilo reverse transcription kit (Invitrogen).

### PCR

To analyze spliced transcripts, 50 ng of cDNA was amplified using 500 nM primers and Q5 High-Fidelity 2X Master Mix (NEB). The primers are listed in the S1 Table. PCR products were separated on a 1% percent gel and post-stained with ethidium bromide. Where indicated, PCR products were gel purified and splice junctions were determined by sequencing.

### Quantitative PCR

50ng cDNA was analyzed in triplicate reactions for three different experiments using an Applied Biosystems QuantStudio 6 Flex real-time PCR thermal cycler (Life Technologies). qPCR was performed using 375 nM primers and SsoAdvanced Universal SYBR Supermix (Bio-Rad). Reaction profiles were setup as follows: initial denature at 95°C for 10 min followed by 40 cycles of 95°C for 15 sec, 63°C for 1 min, 72°C for 30 sec. Melt curves were subsequently performed to ensure proper primer annealing. Relative transcript levels were determined using the threshold cycle method (ΔΔCT) with GAPDH as an endogenous control gene. SETD2 primers are listed in the S1 Table.

### Chromatin immunoprecipitation

ChIP assays were on chromatin prepared from CIN612 cells using 2 μg of anti-H3K36me3 (Abcam), 10 μg of anti-H3.1 (Active Motif) or normal rabbit or mouse IgG, as previously described [57]. Input and immunoprecipitated DNA was quantified in triplicate using the HPV31 qPCR primers listed in the S1 Table. qPCR was performed using an Applied Biosystems QuantStudio 6 Flex real time PCR thermal cycler (Life Technologies). Reaction profiles were setup as follows: initial denature at 95°C for 10 min followed by 40 cycles of 95°C for 15 sec, 63°C for 1 min, 72°C for 30 sec. Melt curves were subsequently performed to ensure proper primer annealing.

## Supporting information

S1 Fig Co-expression of E6 with E7 does not alter SETD2 levels. Whole cell lysates were harvested from uninfected HFKs as well as HFKs retrovirally transduced and stably expressing either wild-type HPV31 E7 or E6/E7 in combination. Western blot analysis was performed using antibodies to SETD2 and p53, with p84 serving as a loading control. Densitometry was performed using Image J software. Relative protein levels for HFK-31 E6 and HFK-31 E6/E7 were quantified by densitometry using ImageJ software and were normalized to the p84 loading control. Values shown are fold differences relative to HFK-31 E7, which is set to 1. Shown is a representative image of three independent experiments.
(TIF)

S2 Fig Depletion of SETD2 minimally affects cellular proliferation. (A) Whole cell lysates were harvested from the same population of CIN612 cells in Fig 3A that were transduced with either control shRNA (shScram) or SETD2 shRNA #2 for 72hr (T0) or for an additional 72hr in high calcium medium to induce differentiation. Western blot analysis was performed using antibodies to cyclin A, cyclin E, RPA32, cyclin B, Cdc25c, CDK1 and CKD2. GAPDH served as a loading control. Ca = calcium. (B) CIN612 cells were seeded at 500,000 cells per 10cm dish. Two days post-seeding, cells were transduced with either control shRNA (shScram) or SETD2 shRNA #2. 72hr post-transduction, cells were harvested and counted. Shown are the averages of two independent experiments. Error bars represent mean ± standard error. Western blot analysis was performed to demonstrate SETD2 knockdown. GAPDH served as a loading control.
(TIF)

S3 Fig SETD2 is necessary for productive viral replication upon differentiation in methylcellulose. CIN612 cells were transiently transduced with either control shRNA (ShScram) or SETD2 shRNA #2 for 72hr. Cells were then either harvested as an undifferentiated sample (T0), or suspended in methylcellulose for 48hr. At the indicated time points, DNA and protein were harvested. DNA was digested with BamHI (non-cutter) and Southern blotting analysis was performed to analyze episome copy number using the HPV31 genome as a probe. Western blot analysis was performed to examine the levels of SETD2. Involucrin and K10 were used as differentiation controls, and GAPDH served as a loading control. MC = methylcellulose. WB = western blot.
(TIF)

S4 Fig SETD2 is necessary for splicing of late L1 RNAs. RNA was extracted from the same pool of undifferentiated (T0) and differentiated (72hr Ca) CIN612 cells shown in Fig 7 that were transiently transduced with either control shRNA (shScram) or SETD2 shRNA #2. Following DNA synthesis, PCR was performed for (A) 30 cycles or (B) 25 cycles using the indicated primers. (A) To analyze splicing across the 877^5552 and 877^3295^5552 junctions, PCR was performed using the E7F (nt 766) and L1R (nt 6595) primer pair. Relative levels of L1a and L1b were determined by performing densitometry using ImageJ software. Values shown indicate the ratio of L1a to L1b at each time point. Splicing across the 3950^5552 junction was determined using the E4F/L1R primer pair and splicing across the 1296^3295 junction was performed using the 1270F/E4R primer pair. GAPDH specific primers were used to control for loading. (B) Levels of E5 were determined using the E7F/E5R primer pair, and levels of spliced E2 were determined using the E7F/E2R primer pair. GAPDH specific primers were used as a loading control. Primer sequences are listed in S1 Table. Ca = calcium. Images are representative of three independent experiments.
(TIF)

S5 Fig Inhibition of ATM kinase activity does not affect the levels of H3.1 on HPV31 DNA. Chromatin was harvested from (A) undifferentiated CIN612 cells treated with DMSO or 10uM of the ATM inhibitor KU55933 for 24hr and (B) CIN612 cells differentiated in high calcium medium for 72hr in the presence of DMSO or 10uM KU55933. ChIP was performed using an antibody to H3.1 using primer pairs indicated in Fig 4A and listed in the S1 Table. Data of ChIP signals from three independent experiments were normalized to 1% of input used. Shown in the fold change in H3.1 binding relative to the first primer set, which is set to one. Error bars represent means ± standard error. Ca = calcium.
(TIF)

S6 Fig ATM activity is required for splicing of late L1 RNAs. RNA was extracted from the same population of CIN612 cells in Fig 8 that were treated with the ATM inhibitor KU55933 or DMSO for 24hr as an undifferentiated sample or for 72hr differentiation in high calcium medium. Following DNA synthesis, PCR was performed for (A) 30 cycles or (B) 25 cycles using the indicated primers. (A) Splicing across the 877^5552 and 877^3295^5552 junctions were analyzed by PCR using the E7F (nt 766) and L1R (nt 6595) primer pair. Relative levels of L1a and L1b were determined by performing densitometry using ImageJ software. Values shown indicate the ratio of L1a to L1b at each time point. Splicing across the 3950^5552 junction was determined using the E4F/L1R primer pair and splicing across the 1296^3295 junction was performed using the 1270F/E4R primer pair. GAPDH specific primers were used to control for loading. (B) Levels of E5 were determined using the E7F/E5R primer pair, and levels of spliced E2 were determined using the E7F/E2R primer pair. GAPDH specific primers were used as a loading control. Primer sequences are listed in S1 Table. Ca = calcium. Images are representative of three independent experiments.
(TIF)

S1 Table List of oligonucleotide primer pairs used in PCR and generation of SETD2 guide RNAs. (DOCX)
