# Supplementary figures and images for: SETD2-dependent H3K36me3 plays a critical role in epigenetic regulation of the HPV31 life cycle
Source: PLoS Pathog. 2018 Oct 12;14(10):e1007367. doi: 10.1371/journal.ppat.1007367 (PMC6200281; doi:10.1371/journal.ppat.1007367)

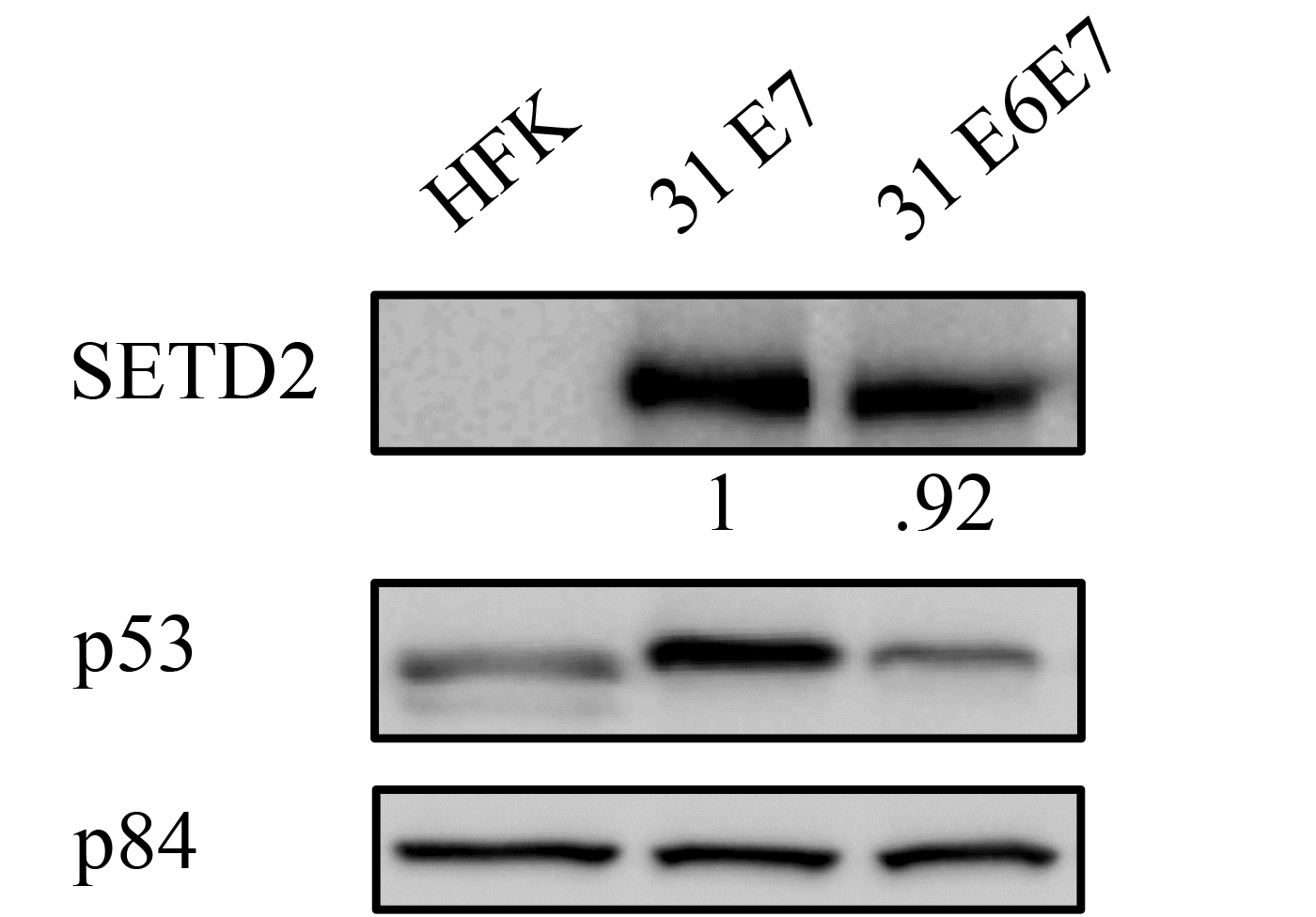

Supplement: S1 Fig — Whole cell lysates were harvested from uninfected HFKs as well as HFKs retrovirally transduced and stably expressing either wild-type HPV31 E7 or E6/E7 in combination. Western blot analysis was performed using antibodies to SETD2 and p53, with p84 serving as a loading control. Densitometry was performed using Image J software. Relative protein levels for HFK-31 E6 and HFK-31 E6/E7 were quantified by densitometry using ImageJ software and were normalized to the p84 loading control. Values shown are fold differences relative to HFK-31 E7, which is set to 1. Shown is a representative image of three independent experiments. (TIF) [file ppat.1007367.s001.tif]

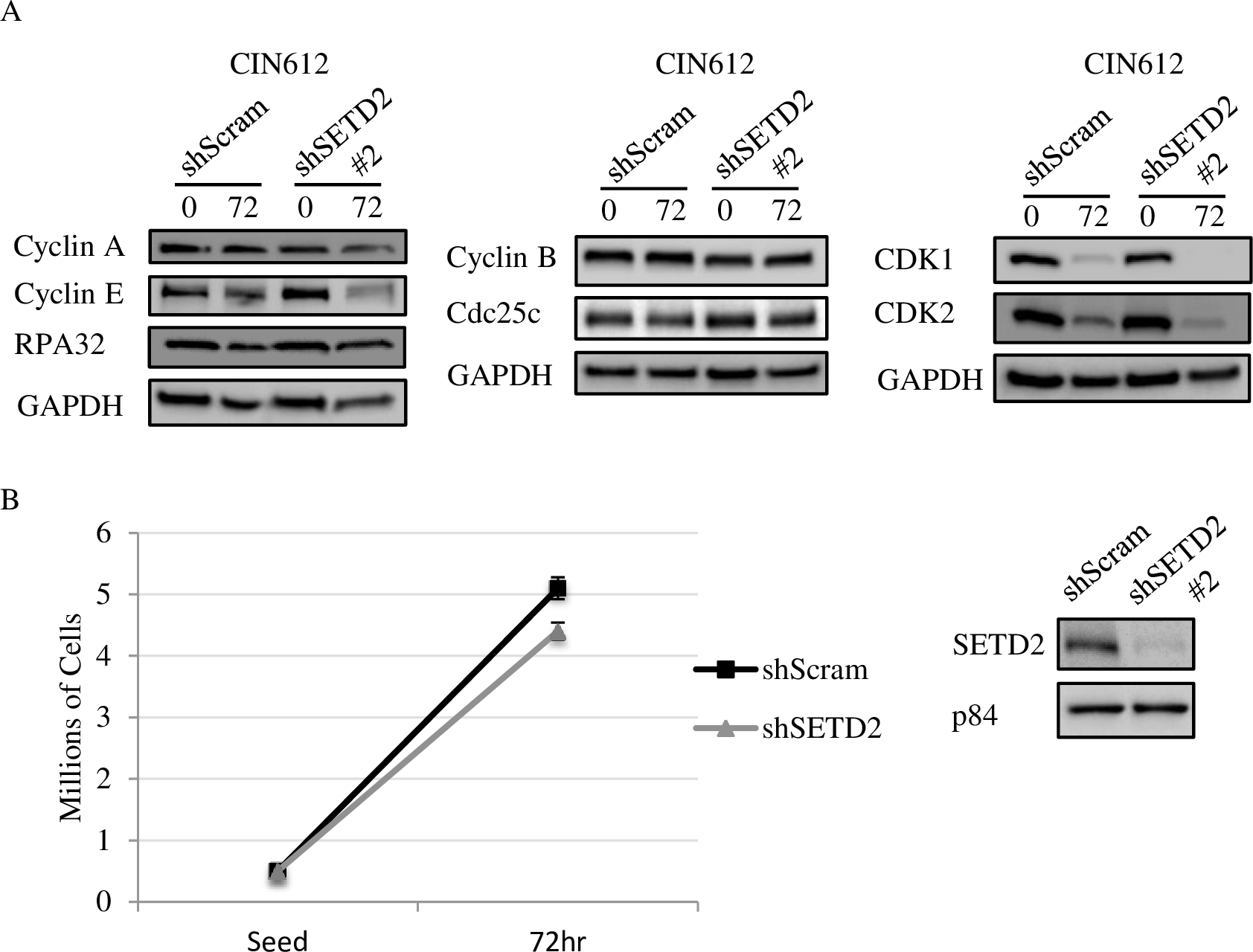

Supplement: S2 Fig — (A) Whole cell lysates were harvested from the same population of CIN612 cells in Fig 3A that were transduced with either control shRNA (shScram) or SETD2 shRNA #2 for 72hr (T0) or for an additional 72hr in high calcium medium to induce differentiation. Western blot analysis was performed using antibodies to cyclin A, cyclin E, RPA32, cyclin B, Cdc25c, CDK1 and CKD2. GAPDH served as a loading control. Ca = calcium. (B) CIN612 cells were seeded at 500,000 cells per 10cm dish. Two days post-seeding, cells were transduced with either control shRNA (shScram) or SETD2 shRNA #2. 72hr post-transduction, cells were harvested and counted. Shown are the averages of two independent experiments. Error bars represent mean ± standard error. Western blot analysis was performed to demonstrate SETD2 knockdown. GAPDH served as a loading control. (TIF) [file ppat.1007367.s002.tif]

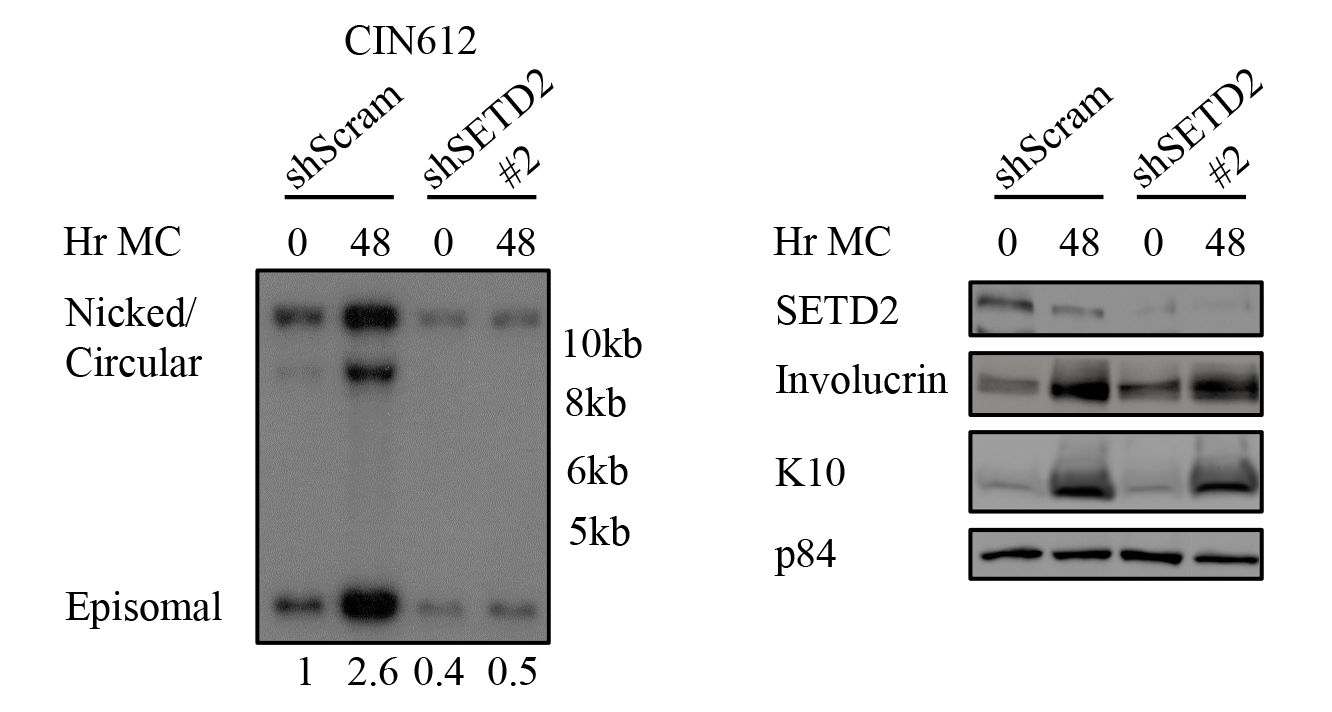

Supplement: S3 Fig — CIN612 cells were transiently transduced with either control shRNA (ShScram) or SETD2 shRNA #2 for 72hr. Cells were then either harvested as an undifferentiated sample (T0), or suspended in methylcellulose for 48hr. At the indicated time points, DNA and protein were harvested. DNA was digested with BamHI (non-cutter) and Southern blotting analysis was performed to analyze episome copy number using the HPV31 genome as a probe. Western blot analysis was performed to examine the levels of SETD2. Involucrin and K10 were used as differentiation controls, and GAPDH served as a loading control. MC = methylcellulose. WB = western blot. (TIF) [file ppat.1007367.s003.tif]

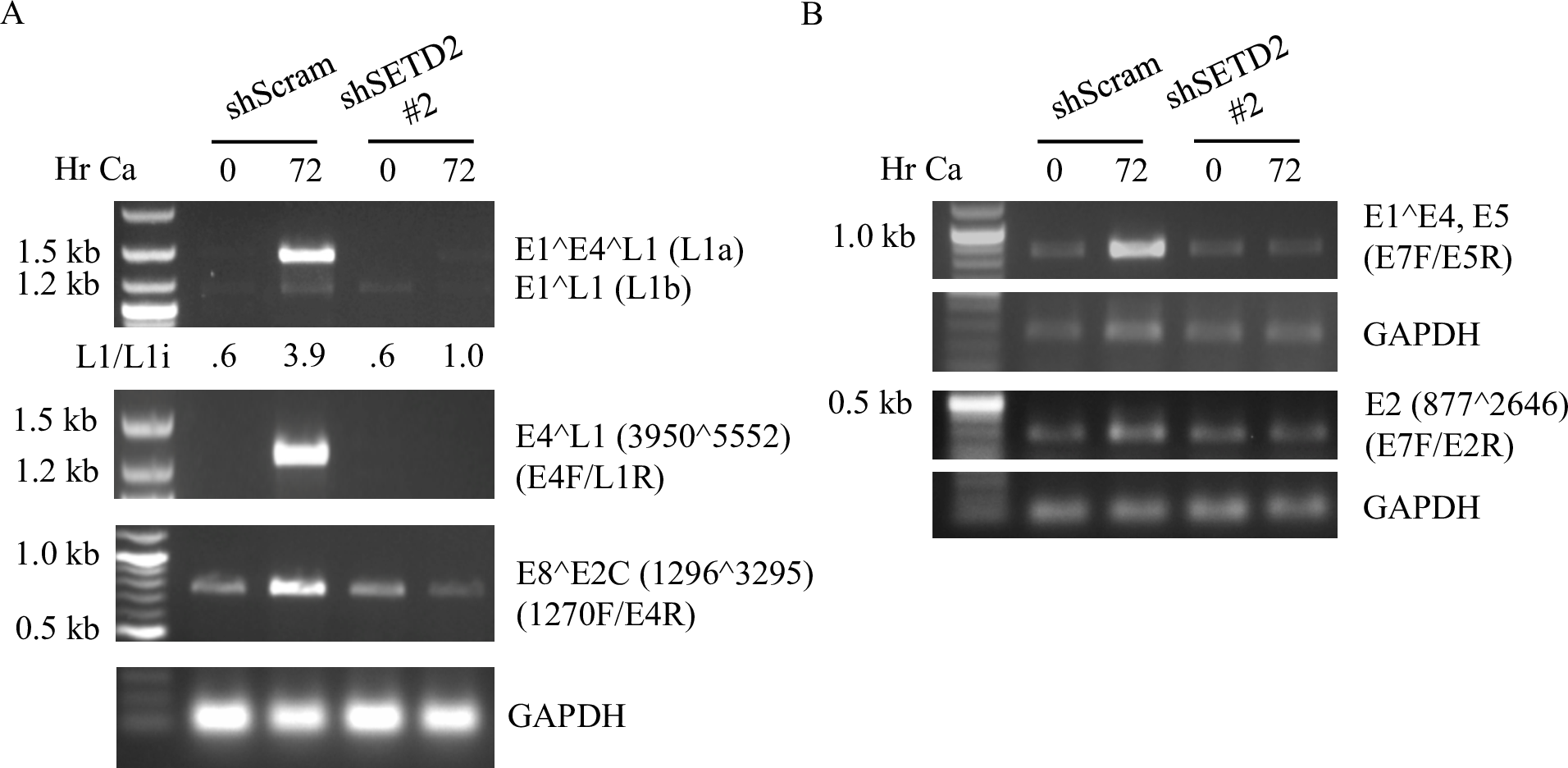

Supplement: S4 Fig — RNA was extracted from the same pool of undifferentiated (T0) and differentiated (72hr Ca) CIN612 cells shown in Fig 7 that were transiently transduced with either control shRNA (shScram) or SETD2 shRNA #2. Following DNA synthesis, PCR was performed for (A) 30 cycles or (B) 25 cycles using the indicated primers. (A) To analyze splicing across the 877^5552 and 877^3295^5552 junctions, PCR was performed using the E7F (nt 766) and L1R (nt 6595) primer pair. Relative levels of L1a and L1b were determined by performing densitometry using ImageJ software. Values shown indicate the ratio of L1a to L1b at each time point. Splicing across the 3950^5552 junction was determined using the E4F/L1R primer pair and splicing across the 1296^3295 junction was performed using the 1270F/E4R primer pair. GAPDH specific primers were used to control for loading. (B) Levels of E5 were determined using the E7F/E5R primer pair, and levels of spliced E2 were determined using the E7F/E2R primer pair. GAPDH specific primers were used as a loading control. Primer sequences are listed in S1 Table. Ca = calcium. Images are representative of three independent experiments. (TIF) [file ppat.1007367.s004.tif]

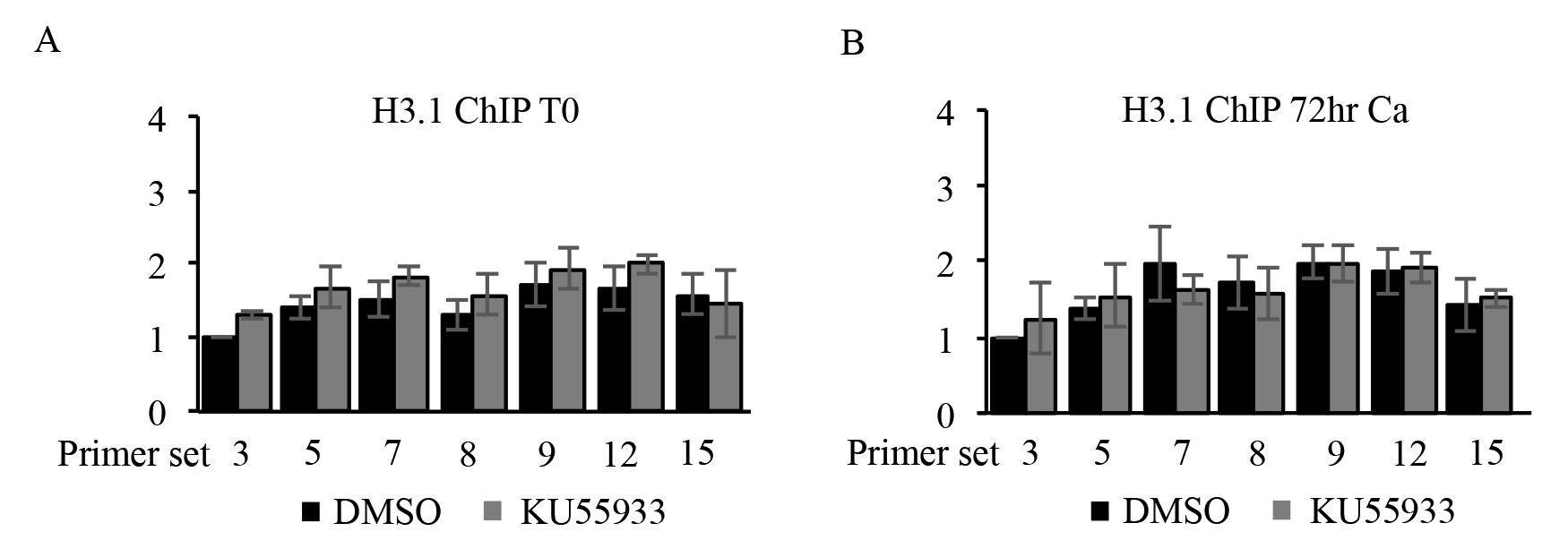

Supplement: S5 Fig — Chromatin was harvested from (A) undifferentiated CIN612 cells treated with DMSO or 10uM of the ATM inhibitor KU55933 for 24hr and (B) CIN612 cells differentiated in high calcium medium for 72hr in the presence of DMSO or 10uM KU55933. ChIP was performed using an antibody to H3.1 using primer pairs indicated in Fig 4A and listed in the S1 Table. Data of ChIP signals from three independent experiments were normalized to 1% of input used. Shown in the fold change in H3.1 binding relative to the first primer set, which is set to one. Error bars represent means ± standard error. Ca = calcium. (TIF) [file ppat.1007367.s005.tif]

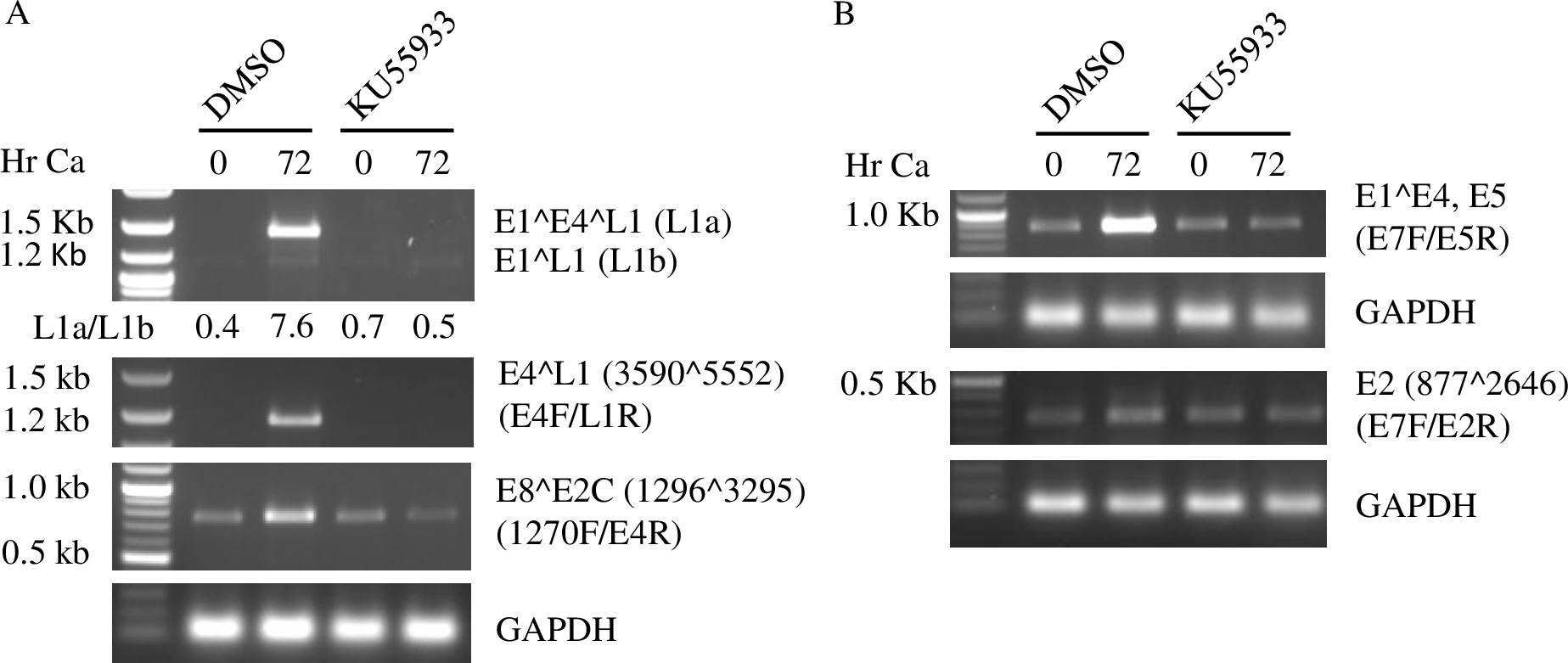

Supplement: S6 Fig — RNA was extracted from the same population of CIN612 cells in Fig 8 that were treated with the ATM inhibitor KU55933 or DMSO for 24hr as an undifferentiated sample or for 72hr differentiation in high calcium medium. Following DNA synthesis, PCR was performed for (A) 30 cycles or (B) 25 cycles using the indicated primers. (A) Splicing across the 877^5552 and 877^3295^5552 junctions were analyzed by PCR using the E7F (nt 766) and L1R (nt 6595) primer pair. Relative levels of L1a and L1b were determined by performing densitometry using ImageJ software. Values shown indicate the ratio of L1a to L1b at each time point. Splicing across the 3950^5552 junction was determined using the E4F/L1R primer pair and splicing across the 1296^3295 junction was performed using the 1270F/E4R primer pair. GAPDH specific primers were used to control for loading. (B) Levels of E5 were determined using the E7F/E5R primer pair, and levels of spliced E2 were determined using the E7F/E2R primer pair. GAPDH specific primers were used as a loading control. Primer sequences are listed in S1 Table. Ca = calcium. Images are representative of three independent experiments. (TIF) [file ppat.1007367.s006.tif]
