# Supplementary material for: SETD2-dependent H3K36me3 plays a critical role in epigenetic regulation of the HPV31 life cycle
Source: PLoS Pathog. 2018 Oct 12;14(10):e1007367. doi: 10.1371/journal.ppat.1007367 (PMC6200281; doi:10.1371/journal.ppat.1007367)
Supplement: S1 Table — (DOCX) [file ppat.1007367.s007.docx]

**S1 Table.** List of oligonucleotide primer pairs used in PCR and generation of SETD2 guide RNAs.

|  | Primers | Orientation | Sequence |
| --- | --- | --- | --- |
| 1 | 92-114 | F | CAAACCTACAGACGCCATGTTCA |
|  | 150-173 | R | GGGTATTTCCAATGCCGAGCTTAG |
| 2 | 215-244 | F | GTTAACAGAAACAGAGGTATTAGATTTTGC |
|  | 274-295 | R | CACACTCCGTGTGGTGTGTCGT |
| 3 | 934-951 | F | GACAGACAGACAGGGGAC |
|  | 987-1006 | R | CAACCATATCCTCCCCAGTA |
| 4 | 1504-1525 | F | GATTGGTGTGTAGCTGCGTTTG |
|  | 1559-1581 | R | CAAACAATATGGTTGCAATAGGG |
| 5 | 2059-2078 | F | ATGTCCATGGGACAGTGGAT |
|  | 2100-2133 | R | CTTTACTATGTCCCTCCAGTCACC |
| 6 | 2578-2597 | F | CCATTTGACAAAAACGGAAA |
|  | 2633-2652 | R | CCACGTCCTTGAGAAAAAGG |
| 7 | 3255-3274 | F | CGGGTGGTCAGGTAATTGTT |
|  | 3309-3329 | R | TTGTAACAATCCCAGCAAAGG |
| 8 | 4174-4192 | F | CGGTCCAAACGCTCTACAA |
|  | 4227-4250 | R | GTACCTGCTGCTTTACATGTTTGA |
| 9 | 4452-4473 | F | CATTGACCCTGTAGGTCCCTTG |
|  | 4510-4528 | R | GGGCACCAACATCAACAAT |
| 10 | 5105-5124 | F | CTATTGGTGCAAGGGTGCAT |
|  | 5158-5180 | R | GGTTGCATTTCAATACTTTCACC |
| 11 | 5407-5428 | F | CATGCACCTACACAGGTTTTCC |
|  | 5464-5483 | R | TCACCCCCATCAACAAAAAT |
| 12 | 5591-5611 | F | CCACCTGTCCCAGTGTCTAAA |
|  | 5643-5665 | R | GCCTGCGTGATAATATATGTTGG |
| 13 | 6098-6117 | F | CCTGGTGATTGTCCTCCATT |
|  | 6153-172 | R | TCCAAAGCCTGTATCAACCA |
| 14 | 6521-6540 | F | ATTTGTTGGGGCAATCAGTT |
|  | 6574-6595 | R | TGCAGCACAAACAGACATATTG |
| 15 | 6967-6986 | F | GGCAGGATATAGGGCACGTC |
|  | 7021-7041 | R | GCTGGTGTAGTGGTAGATGCTG |
| 16 | 7336-7356 | F | GTTCCTGCTCCTCCCAATAG |
|  | 7385-7411 | R | GTACAACTTTTACTATGGCGTGACAC |
| 17 | 7708-7734 | F | TCCTACACACCTTAAACTGCTTTTAGG |
|  | 7764-7784 | R | GCAAAAGCCAGCACTGCAATC |
|  | SETD2 | F | CTTTCTGTCCCACCCCTGTC |
|  | SETD2 | R | CCTTGCACCTCTGATGGCTT |
| sgSETD2 | 017_Array | F | TAACTTGAAAGTATTTCGATTTCTTGGCTTTATATATCTTGTGGAAAGGACGAAACACCG |
| sgSETD2 | 017_Array | R | ACTTTTTCAAGTTGATAACGGACTAGCCTTATTTTAACTTGCTATTTCTAGCTCTAAAAC |
| HPV31 121 | 121-144 | F | CTGCAGAAAGACCTCGGAAATTGC |
| HPV31 E4 | 3432-3452 | R | TTCTGTGCTCTGGCTCTGTTC |
| HPV31 295 | 275-295 | R | CACACTCCGTGTGGTGTGTCG |
| HPV31 E4 | 3353-3373 | F | ACCACATCGAATTCCAAAACC |
| HPV31 E7 | 633-652 | R | GCTGTCGGGTAATTGCTCAT |
| HPV31 E5 | 4032-4052 | R | AGATGCATGTGTATGAATTAC |
| HPV31 E8^E2 | 1270-1290 | F | GAAGTGGAAACGCAGCAGATG |
| HPV31 E8^E2 | 4031-4050 | R | ATGCATGTGTATGAATTACA |
| HPV31 E2 | 2783-2807 | R | CAAGTCGAATATGTTTCCAATAGTC |
| HPV31 E7 | 766-789 | F | ACAGAGCACACAAGTAGATATTCG |
| HPV31 L1 | 6574-6595 | R | TGCAGCACAAACAGACATATTG |
